# Gastrointestinal symptoms of long COVID-19 related to the ectopic colonization of specific bacteria that move between the upper and lower alimentary tract and alterations in serum metabolites

**DOI:** 10.1186/s12916-023-02972-x

**Published:** 2023-07-19

**Authors:** Deyu Zhang, Siyuan Weng, Chuanchao Xia, Yuqing Ren, Zaoqu Liu, Yudi Xu, Xiaoli Yang, Ruhao Wu, Lisi Peng, Liqi Sun, Jiaqi Zhu, Xuesong Liang, Yin Jia, Huaizhou Wang, Qian Chen, Dongtian Liu, Yi Chen, Honglei Guo, Xinwei Han, Zhendong Jin, Cui Chen, Xia Yang, Zhaoshen Li, Haojie Huang

**Affiliations:** 1 Department of Gastroenterology, Changhai Hospital, Naval Medical University, Shanghai, 200433 China; 2 Department of Interventional Radiology, The First Affiliated Hospital of Zhengzhou University, Zhengzhou, 450052 Henan China; 3 Department of Respiratory and Critical Care Medicine, The First Affiliated Hospital of Zhengzhou University, Zhengzhou, 450052 Henan China; 4 Department of Neurology, The First Affiliated Hospital of Zhengzhou University, Zhengzhou, 450052 Henan China; 5 Department of Gastroenterology, General Hospital of Ningxia Medical University, Ningxia Medical University, Yinchuan, 750003 Ningxia China; 6 Department of Cardiology, Changhai Hospital, Naval Medical University, Shanghai, 200433 China; 7 Department of Infectious Diseases, Changhai Hospital, Naval Medical University, Shanghai, 200433 China; 8 Department of Laboratory Medicine, Changhai Hospital, Naval Medical University, Shanghai, 200433 China; 9 Department of Rheumatology and Immunology, Changhai Hospital, Naval Medical University, Shanghai, 200433 China; 10 Shanghai Foreign Language School Affiliated to Shanghai International Studies University, Shanghai, 200433 China; 11 Department of Gastroenterology, No. 905 Hospital of The Chinese People’s Liberation Army, Shanghai, 200050 China

**Keywords:** Coronavirus, Gut microbiome, Microbial functions, Serum metabolomics

## Abstract

**Background:**

Since the coronavirus disease 2019 (COVID-19) outbreak, many COVID-19 variants have emerged, causing several waves of pandemics and many infections. Long COVID-19, or long-term sequelae after recovery from COVID-19, has aroused worldwide concern because it reduces patient quality of life after rehabilitation. We aimed to characterize the functional differential profile of the oral and gut microbiomes and serum metabolites in patients with gastrointestinal symptoms associated with long COVID-19.

**Methods:**

We prospectively collected oral, fecal, and serum samples from 983 antibiotic-naïve patients with mild COVID-19 and performed a 3-month follow-up postdischarge. Forty-five fecal and saliva samples, and 25 paired serum samples were collected from patients with gastrointestinal symptoms of long COVID-19 at follow-up and from healthy controls, respectively. Eight fecal and saliva samples were collected without gastrointestinal symptoms of long COVID-19 at follow-up. Shotgun metagenomic sequencing of fecal samples and 2bRAD-M sequencing of saliva samples were performed on these paired samples. Two published COVID-19 gut microbiota cohorts were analyzed for comparison. Paired serum samples were analyzed using widely targeted metabolomics.

**Results:**

Mild COVID-19 patients without gastrointestinal symptoms of long COVID-19 showed little difference in the gut and oral microbiota during hospitalization and at follow-up from healthy controls. The baseline and 3-month samples collected from patients with gastrointestinal symptoms associated with long COVID-19 showed significant differences, and ectopic colonization of the oral cavity by gut microbes including 27 common differentially abundant genera in the Proteobacteria phylum, was observed at the 3-month timepoint. Some of these bacteria, including Neisseria, Lautropia, and Agrobacterium, were highly related to differentially expressed serum metabolites with potential toxicity, such as 4-chlorophenylacetic acid, 5-sulfoxymethylfurfural, and estradiol valerate.

**Conclusions:**

Our study characterized the changes in and correlations between the oral and gut microbiomes and serum metabolites in patients with gastrointestinal symptoms associated with long COVID-19. Additionally, our findings reveal that ectopically colonized bacteria from the gut to the oral cavity could exist in long COVID-19 patients with gastrointestinal symptoms, with a strong correlation to some potential harmful metabolites in serum.

**Supplementary Information:**

The online version contains supplementary material available at 10.1186/s12916-023-02972-x.

## Background

It has been 3 years since the outbreak of coronavirus disease 2019 (COVID-19), which is caused by severe acute respiratory syndrome coronavirus 2 (SARS-CoV-2), and there is scientific consensus that COVID-19 can affect organs other than the respiratory system, especially the gastrointestinal (GI) tract [1–4]. Many studies have illustrated that the gut microbiome is altered during COVID-19, including some genera in the Lachnospiraceae and Ruminococcaceae families and some genera that function as opportunistic pathogens, including Streptococcus, Rothia, Veillonella, and Actinomyces [5–7]. Other studies have demonstrated the presence of changes in the oral microbiome associated with COVID-19, including decreased microbial diversity, an increased proportion of lipopolysaccharide-producing bacteria, and a decreased proportion of butyric acid-producing bacteria [8–10].

The intestinal microecology includes not only intestinal bacteria but also a large number of viruses, fungi, and a small amount of archaea and protozoa. The balance between these microbes plays an important role in maintaining health. Viral infection not only affects the intestinal flora but also stimulates the immune response of the intestine, thus affecting health [11]. The GI tract is considered the body’s largest immune organ, and the gut microbiota can control host immunity, combat pathogens, and assist in nutrient metabolism [12]. Numerous variables, including genes, diet, lifestyle, illness, and aging, can dynamically change the composition of the gut microbiota [13, 14]. Immune-mediated inflammatory and autoimmune diseases are frequently related to gut dysbiosis and a reduction in microbial diversity [15, 16]. Studies have shown that respiratory infections are linked to both compositional and functional alterations of the gut microbiota due to the critical crosstalk between gut microorganisms and the pulmonary system, which comprises the “gut-lung axis” [17]. Interruption of the gut microbiota may have a detrimental effect on the recruitment of immune cells to the lung, which may then increase the risk of developing respiratory tract infections [18]. In particular, COVID-19 has been linked to changes in the microbiome and dysfunction of the gut barrier in human studies, which may promote the translocation of bacterial products and toxins into the circulatory system and aggravate the inflammatory response throughout the body [19]. The angiotensin-converting enzyme 2 (ACE2) receptor is highly expressed in the intestines and plays an important role in maintaining gut health [20]. Infection with SARS-CoV-2 could impair the normal expression of ACE2, which might result in several adverse outcomes, including GI symptoms as well as dysbiosis of the gut microbiota [21]. Alternatively, intestinal infection may directly damage the intestinal structure, destroy the intestinal epithelial barrier, and promote intestinal inflammation [22].

Long COVID-19, also known as post-COVID-19 syndrome, is a clinical spectrum of conditions including various physical and neuropsychiatric symptoms lasting for several months after the test for the virus becomes negative, without an alternative explanation [23, 24]. Long COVID-19 has been found to be related to the microbiota in the alimentary tract. Specifically, some reports illustrated that the alteration of the oral and gut microbiome could persist after recovery [25, 26], and long COVID-19 is associated with specific and sustained changes in the oral and gut microbiomes of patients [27–31]. However, these studies have some limitations. First, these studies exhibit high heterogeneity, mainly due to the high variety of severe symptoms that can occur after patients are infected by primitive COVID-19 [32]. Second, all of these previous studies predefined different symptoms of long COVID-19 as one disease with different manifestations [25–31]. Long COVID-19 includes a variety of symptoms related to different organs, and the pathogenesis of these symptoms may be attributed to the dysfunction of different organs [33]. Third, all of the recent studies that investigated changes in oral bacteria have utilized 16S sequencing, which has low resolution in the identification of bacterial species [25–31, 34]. Fourth, these studies performed analyses of primitive COVID-19 infection cases, including a large number of severe cases; the results exhibit nonnegligible bias and cannot be used to characterize the current pandemic [25–31, 35]. The variant of SARS-CoV-2 involved in the current pandemic has become Omicron, which is highly infectious and associated with a low incidence of severe cases [36], and some reports have also indicated that a considerable proportion of patients who recover from mild COVID-19, which ranges between 10 and 35%, experience persistent symptoms [37]. Moreover, these studies have focused on the microbiome associated with one specific organ [25–31, 38]. However, the oral cavity and gut are the two largest microbial ecosystems in the upper and lower alimentary tract, respectively. Recent studies have demonstrated that oral-to-gut and gut-to-oral microbial transmission can regulate pathogenesis, indicating the presence of the oral–gut microbiome axis [39, 40]. Therefore, it is necessary to comprehensively explore the characteristics of the oral and gut microbiomes in patients with GI symptoms associated with long COVID-19.

To achieve this goal and to obtain data that can be compared to gut microbiome data acquired through metagenomics, the resolution of the methods used to characterize the oral microbiome needs to be higher than that of the current 16S sequencing method. 2bRAD sequencing for microbiome (2bRAD-M) analysis is a novel sequencing approach that is used to study the microbiome. 2bRAD-M has proven to be useful in profiling the low-biomass microbiome at the species level with much higher fidelity than 16S sequencing [41]. However, to the best of our knowledge, 2bRAD-M has not been used to assess the microbial characteristics of COVID-19.

In this study, we prospectively collected oral, fecal, and serum samples from 983 antibiotic treatment-naïve patients with mild COVID-19 and followed them up from hospitalization until 3 months after discharge. Then, we collected oral and fecal samples from 45 patients with GI symptoms of long COVID-19 during the 3-month follow-up and characterized the alterations and dynamics of the oral and gut microbiome in these patients between hospitalization and follow-up using 2bRAD-M and metagenomic analysis. We also collected and sequenced the oral and fecal samples from 8 patients without GI symptoms of long COVID-19 during the 3-month follow-up and analyzed data obtained from two former publications on the gut microbiota analysis of COVID-19 patient cohorts, including a cohort from Hong Kong and one from Shanghai, to compare long COVID-19 patients with patients without long COVID-19. Then, we performed targeted metabolomic analysis of serum samples to identify correlations between altered bacteria and serum metabolites.

## Methods

### Study population

This study was approved by the Shanghai Changhai Hospital Ethics Committee (reference number 2022–058), and all patients provided written informed consent. A total of 983 COVID-19 patients were recruited at Changhai Hospitals in Shanghai between April 2022 and June 2022. The inclusion criteria included SARS-CoV-2 reverse transcription polymerase chain reaction positivity based on respiratory specimens; hospitalization; and no probiotic, prebiotic, or antibiotic use in the 3 months before enrollment (Table 1). All of these enrolled patients were vaccinated by one kind of COVID-19 vaccine (CanSino Vac, Sinovac, or CoronaVac) in 1 year. The criteria for mild COVID-19 were fever, cough, or diarrhea, without radiographic indications of pneumonia [42]. Standardized meals were provided to the COVID-19 patients during hospitalization, and the dietary composition and timing of the meals were consistent with the habitual diet commonly consumed by individuals in Shanghai. The oral, fecal, and serum samples of these 983 patients were collected during hospitalization.

**Table 1:** Clinical characteristics of the enrolled COVID-19 patients

Male, *n* (%)	486(49.4)
Female, *n* (%)	497(50.6)
Age, year (min–max)	38.3(23–68)
Hypertension, *n* (%)	103(10.5)
Diabetes mellitus, *n* (%)	52(5.3)
Hyperlipidaemia, *n* (%)	63(6.4)
Duration of hospitalization (days)	9.6(4–21)
Symptoms at admission, *n* (%) Fever	752(76.5)
Gastrointestinal symptoms, *n* (%) Diarrhea	103(10.5)
Respiratory symptoms, *n* (%)	
Cough	531(54)
Rhinorrhea (runny nose)	642 (65.3)
Long COVID rate, *n* (%)	
Extra-gastrointestinal manifestations	54(5.5)
Gastrointestinal manifestations	45(4.6)

After discharge, enrolled COVID-19 patients were advised to continue a diverse and standard Chinese diet that was consistent with the daily diet consumed by individuals in Shanghai. Additionally, patient follow-up was performed 3 months after discharge by telephone. GI symptoms of long COVID-19 were defined as at least one persistent GI symptom, including decreased appetite, diarrhea, abdominal pain, taste disorder, emaciation, and xerostomia, which could not be explained by an alternative diagnosis 3 months after clearance of SARS-CoV-2. After 3 months of follow-up, 45 patients with the above symptoms were identified as patients with GI symptoms of long COVID-19, and they were invited to return to the hospital (Tables 2 and 3). None of these patients had GI disorders before the infection, and none of them received probiotics, prebiotics, or antibiotics during follow-up. Forty-five paired fecal, saliva, and 33 serum samples from these patients were collected after informed consent was obtained when they returned to the hospital. Moreover, these patients underwent symptomatic treatment with specific drugs. For example, Clostridium butyricum tablets were given to patients with decreased appetite, diarrhea, or emaciation. Compound vitamin B tablets and Niuhuangjiedu tablets (Chinese medicine) were given to patients with taste disorders or xerostomia. Pinaverium bromide tablets were suggested for patients with abdominal pain.

**Table 2:** Clinical characteristics of the enrolled patients with gastrointestinal symptoms of long COVID-19 after 3 months of follow-up and healthy controls

Characteristic	COVID-19	Healthy control
Male, *n* (%)	27(60)	13(52)
Female, *n* (%)	18(40)	12(48)
Age, year	40.5(23–68)	35.6(23–56)
Hypertension, *n* (%)	10(22.2)	3(12)
Diabetes mellitus, *n* (%)	5(11.1)	2(8)
Hyperlipidaemia, *n* (%)	7(15.5)	4(16)
Duration of hospitalization (days)	9.6(5–17)	
Symptoms at admission, *n* (%) Fever	31(68.9)	
Gastrointestinal symptoms, *n* (%)		
Diarrhea	6(13.3)	
Taste disorder	28(62.2)	
Respiratory symptoms, *n* (%)		
Cough	17(37.8)	
Rhinorrhea (runny nose)	23(51.1)	
Gastrointestinal symptom of Long COVID-19, *n* (%)		
Decreased appetite	30(66.7)	
Diarrhea	17(37.8)	
Xerostomia	10(22.2)	
Taste disorder	5(11.1)	
Emaciation	5(11.1)	
Abdominal pain	3(6.7)

**Table 3:** Details of the disease occurrence in enrolled patients with gastrointestinal symptoms of long COVID-19 at the 3-month follow-up

Characteristic	After negative	In 1st month	In 1st–2nd month
Male, *n* (%)	17(63)	9(56.3)	1(50)
Female, *n* (%)	10(37)	7(43.7)	1(50)
Age, year (min–max)	46.2(23–68)	35.6(27–53)	35(24,46)
Hypertension, *n* (%)	7(25.9)	2(12.5)	1(50)
Diabetes mellitus, *n* (%)	5(18.5)	0(0)	0
Hyperlipidemia, *n* (%)	6(22.2)	1(6.3)	0
Duration of hospitalization (days)	12.6(8–17)	8.2(5–13)	9(7,11)
Symptoms at admission, *n* (%) Fever	21(77.8)	9(56.3)	1(50)
Gastrointestinal symptoms, *n* (%)			
Diarrhea	6(22.2)	0	0
Taste disorder	18(66.7)	8(50)	0
Respiratory symptoms, *n* (%)			
Cough	10 (37)	5(31.3)	2(100)
Rhinorrhea (runny nose)	18(66.6)	5(31.3)	0
Gastrointestinal symptom of Long COVID, *n* (%)			
Decreased appetite	24 (88.9)	5(31.3)	1(50)
Diarrhea	9(33.3)	7(43.8)	1(50)
Xerostomia	4 (14.8)	4(25)	0
Taste disorder	5(18.5)	0	0
Emaciation	3(11.1)	2(12.5)	0
Abdominal pain	1(3.7)	1(6.25)	1(50)

Additionally, after 3 months of follow-up, another group of 8 patients without GI symptoms were invited to return to the hospital (Table 4). Eight paired fecal and saliva from these patients were collected after informed consent was obtained when they returned to the hospital.

**Table 4:** Details of the enrolled patients without gastrointestinal symptoms of long COVID-19 at the 3-month follow-up

Characteristic	
Male, *n* (%)	4(50)
Female, *n* (%)	4(50)
Age, year (min–max)	43.8(28–57)
Hypertension, *n* (%)	1(12.5)
Diabetes mellitus, *n* (%)	1(12.5)
Hyperlipidemia, *n* (%)	1(12.5)
Duration of hospitalization (days)	11.4(6–15)
Symptoms at admission, *n* (%)	
Fever	7(87.5)
Diarrhea	2(25)
Taste disorder	4(50)
Respiratory symptoms, *n* (%)	
Cough	4(50)
Rhinorrhea (runny nose)	6(75)
Other long COVID symptoms (without gastrointestinal symptoms), *n* (%)	6(75)
Insomnia	2(25)
Fatigue	4(75)

Twenty-five healthy controls were also recruited between June 2022 and August 2022 from citizens in Shanghai, and none of the participants used probiotics, prebiotics, or antibiotics in the 3 months before enrollment. We selected controls matched for age and sex with similar comorbidities and standard dietary patterns. All of these healthy controls were also vaccinated by one kind of COVID-19 vaccine (CanSino Vac, Sinovac, or CoronaVac) in 1 year. Twenty-five paired fecal, saliva, and serum samples from these healthy controls were collected after informed consent was obtained.

Altogether, 983 oral and fecal samples were initially collected during hospitalization. Forty-five patients were identified to exhibit GI symptoms of long COVID-19 during the 3-month follow-up (Table 1). Then, 45 saliva samples and fecal samples and 33 serum samples were collected from these patients, who were designated as the follow-up group. Eight saliva samples and paired fecal samples are collected from patients without GI symptoms during the 3-month follow-up. Initial oral, fecal, and serum samples were collected from the original 983 hospitalized patients, who were designated as the mild group. Saliva, fecal, and serum samples were also collected from 25 healthy controls, who were designated as the normal group. The samples obtained from these three groups were collected and subjected to sequencing.

### Sample collection

For the collection of oral samples, all of the participants were required to avoid eating, drinking, and brushing their teeth 2 h before taking samples and then rinse their mouths with sterile water. Unstimulated saliva was collected in 90 mm*15 mm culture dishes (BKMAM bio company, China) and subsequently transported within 30 min from the hospital to the laboratory in an ice bag using insulating polystyrene foam containers. Then, the saliva was transferred to sterile 1.5-mL microcentrifuge tubes, frozen at − 80 °C until further processing (no more than 6 months) and thawed immediately prior to DNA extraction.

Fecal samples were collected using a swab containing bacterial DNA Locker (Youkang, Nanjing, China) and subsequently transported within 30 min from the hospital to the laboratory in an ice bag using insulating polystyrene foam containers. In the laboratory, the swabs were immediately stored at − 80 °C and thawed immediately prior to DNA extraction (no more than 6 months).

For collection of serum samples, peripheral vein blood was collected from all recruited participants under a fasting state with vacuous tubes and then separated into serum through centrifugation at 3000 rpm and 4 °C for 10 min. Each aliquot of the obtained serum samples was placed at − 80 °C and stored until further procedure (no more than 6 months).

### DNA isolation and library construction for metagenomic sequencing

Total DNA from fecal samples was isolated using a QIAamp® Fast DNA Stool Mini Kit (Qiagen, Hilden, Germany) following the manufacturer’s instructions. DNA concentration and integrity were assessed by a NanoDrop2000 spectrophotometer (Thermo Fisher Scientific, Waltham, MA, USA) and agarose gel electrophoresis, respectively. DNA was fragmented by S220 Focused-ultrasonicators (Covaris, USA) and cleaned by Agencourt AMPure XP beads (Beckman Coulter Co., USA). Then, the libraries were constructed using the TruSeq Nano DNA LT Sample Preparation Kit (Illumina, San Diego, CA, USA) according to the manufacturer’s instructions. Metagenome sequencing and analysis were conducted by OE Biotech Co., Ltd. (Shanghai, China). The details of metagenome sequencing and bioinformatics analysis are shown in the online Additional file 1: supplementary material [41, 43–51].

### Downloading of metagenomic data obtained from fecal samples collected from mild COVID-19 patients in previous studies

Metagenomic data were obtained from the NODE (https://www.biosino.org/node/project/detail/OEP002590) and SRA (https://www.ncbi.nlm.nih.gov/bioproject/PRJNA689961) databases. The fecal metagenomic data obtained from the Shanghai cohort and Hong Kong cohort were downloaded. The Shanghai cohort [19] dataset included data from 62 patients with mild COVID-19 and 8 healthy controls, and the Hong Kong cohort dataset included data from 48 patients with mild disease, 13 follow-up patients, and 70 healthy controls [25]. The details of the metagenomic bioinformatics analysis are shown in the Additional file 1: supplementary material.

### DNA isolation and library construction and sequencing for 2bRAD-M sequencing

Total DNA from oral samples was isolated using a QIAamp® Fast DNA Stool Mini Kit (Qiagen, Hilden, Germany) following the manufacturer’s instructions. DNA concentration and integrity were assessed by a NanoDrop2000 spectrophotometer (Thermo Fisher Scientific, Waltham, MA, USA) and agarose gel electrophoresis, respectively. DNA was fragmented by S220 Focused-ultrasonicators (Covaris, USA) and cleaned by Agencourt AMPure XP beads (Beckman Coulter Co., USA). The 2bRAD-M library preparation method basically followed the original protocol developed by Wang et al. [52] with few modifications. DNA (1 pg–200 ng) was digested with 4 U of the enzyme BcgI (NEB, USA) for 3 h at 37 °C. Subsequently, adaptors were ligated to the DNA fragments. The ligation reaction was performed by combining 5 µl of digested DNA with 10 µl of a ligation master mix containing 0.2 µM each of two adaptors and 800 U T4 DNA ligase (NEB, USA). Ligation was carried out at 4 °C for 12 h. Then, the ligation products were amplified, and the PCR products were subjected to 8% polyacrylamide gel electrophoresis. Bands of approximately 100 bp were excised from the polyacrylamide gel, and the DNA was diffused from the gel in nuclease-free water for 12 h at 4 ℃. Sample-specific barcodes were introduced by PCR with platform-specific barcode-bearing primers. Each 20 µl PCR sample contained 25 ng of gel-extracted PCR product, 0.2 µM of each primer, 0.3 mM dNTP, 1 × Phusion HF buffer, and 0.4 U Phusion high-fidelity DNA polymerase (NEB, USA). PCR products were purified using a QIAquick PCR purification kit (Qiagen, Germany) and then subjected to sequencing using the Illumina Nova PE150 platform. 2bRAD-M was carried out at Qingdao OE Biotech Co., Ltd. (Qingdao, China). Then, we performed bioinformatics analysis, and the details are shown in the Additional file 1: supplementary material.

### Full-spectrum metabolomics analysis

After informed consent was obtained, fasting blood samples from all 983 enrolled patients were collected during hospitalization in the morning hours. After 3 months of follow-up, 45 long COVID-19 patients with gastrointestinal symptoms were identified, and 33 of them agreed to return to the hospital and consented to provide blood samples. The diagnostic criteria that were used are the same as those described above. Blood samples were obtained from all 25 healthy controls. The blood collection procedure was standardized and uniform. All collected blood samples were prepared by centrifugation at 3000 rpm for 10 min, and the supernatant was carefully collected to obtain serum. Serum samples were inactivated using a 60 ℃ water bath for 10 min, shaken vigorously, dried in a biosafety hood, and then stored at − 80 °C. First, all samples were thawed slowly at 4 °C. The sample information for all 33 patients and healthy volunteers is presented in Additional file 2: Table S1. Then, the serum metabolites were evaluated using ultra-performance liquid chromatography (UPLC) coupled with tandem mass spectrometry (MS/MS) (ExionLCAD coupled to a QTRAP spectrometer) (https://sciex.com.cn/). The liquid-phase conditions were as follows: (1) chromatographic separation using a Waters Acquity UPLC HSS T3 column (1.8 µm particle size, 100 mm × 2.1 mm; (2) analyte elution on a mobile phase with ultrapure water (0.1% formic acid) for phase A and acetonitrile (0.1% formic acid) for phase B; (3) elution gradients of water/acetonitrile (95:5 V/V) at 0 min, 10:90 V/V at 11.0 min, 10:90 V/V at 12.0 min, 95:5 V/V at 12.1 min, and 95:5 V/V at 14.0 min; and (4) a flow rate of 0.4 ml/min, a column temperature of 40 °C, and an injection volume of 2 ml. The MS/MS conditions were as follows: 500 °C; electrospray ionization; 5500 V (positive) and − 4500 V (negative); 55 psi, ion gas source I; 60 psi, gas source II; 25 psi, curtain gas; and high collision-activated dissociation parameters. The ions were scanned and detected according to an optimized decluttering potential and collision energy. The ions were subjected to qualitative identification based on the retention time, daughter–parent ion pair information, and secondary spectral data obtained from the detected substances using the MetWare database (http://www.metware.cn/). The analytes were quantified using the QTRAP multiple-reaction monitoring mode. After data were collected from different samples, the area under the peak was scored separately for the chromatographic peaks of the extracted ions obtained from all metabolites. Finally, score correction for the chromatographic peaks of the same metabolite in different specimens was performed. Then, the results were verified through quality control.

### Identification of differential metabolites

The metabolite data were subjected to different univariate and multivariate analyses to identify differentially expressed metabolites. PCA and orthogonal partial least squares-discriminant analysis (OPLS-DA) were performed using R (v. 4.1.3) to reduce data dimensionality and verify the separation trends between groups. OPLS-DA combines orthogonal signal correction (OSC) and the partial least squares-discriminant analysis (PLS-DA) method. In this study, OPLS-DA was performed to decompose the X matrix information (metabolic profile) into Y (group) correlations and irrelevance by OSC and PLS-DA, thereby enabling differential metabolites to be screened by removing unrelated differences. The relative levels of the differentially expressed metabolites were normalized and centralized, and K-means clustering (K-means) analysis was performed to investigate the change trends of the relative metabolite levels in different samples. The OPLS-DA results yielded variable importance in projection (VIP) values for each metabolite, and only those with VIP clustering (based on K-mean values in the permutation test) were used to evaluate the OPLS-DA model. In addition, metabolites with levels that had a fold change of − 1.5 and a *p* value ≤ 1.05 were identified as significantly differentially expressed.

### Statistical analysis

Statistical analyses were performed using R software (v. 4.1.3). The Shapiro‒Wilk test and Bartlett test were performed to assess the normality and homogeneity of variance of the data, respectively. For data that met both normal distribution and homogeneity of variance, we used analysis of variance (ANOVA) or Student’s *t* test; otherwise, Kruskal‒Walli’s test and Wilcoxon test were used. When multiple group comparisons were statistically significant, further two group comparisons were performed, and *P* values were corrected by Bonferroni’s method. Correlation analysis of two continuous variables was performed by the nonparametric Spearman correlation test. We used the linear discriminant analysis (LDA) effect size (LEfSe) method to identify the group-specific biological function of microbes in fecal samples. Differences with a *p* value < 0.05 (two-sided) were considered statistically significant.

## Results

### Study design

**Fig. 1:**
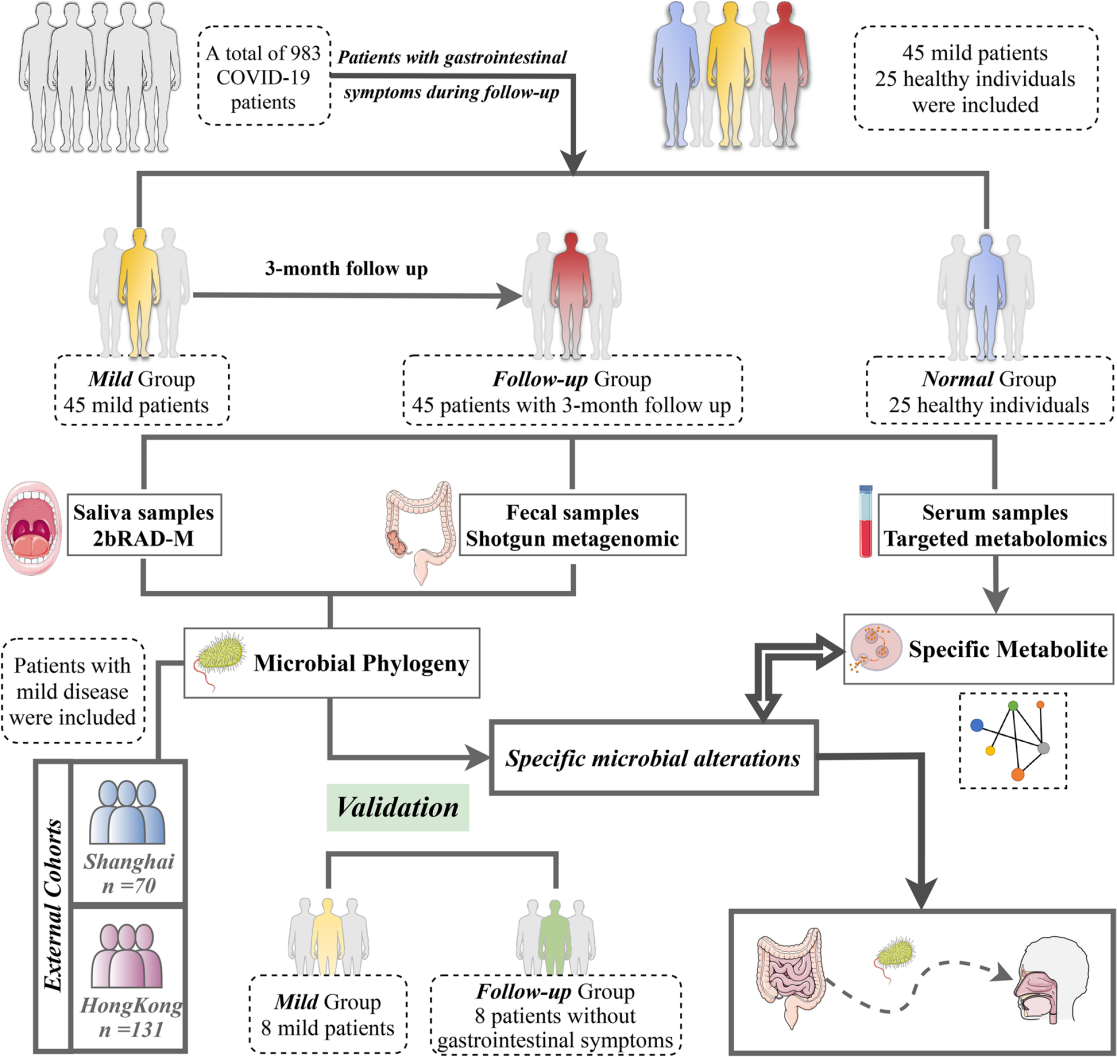
Study design and flow chart

As shown in Fig. 1, this study consisted of two parts. First, to explore the differences in the gut microbiota between mild COVID-19 patients during hospitalization and at follow-up and healthy controls, two cohorts of metagenomic data were collected, and samples from mild patients or healthy controls were filtered and analyzed; these data included data obtained from a Hong Kong cohort comprising 70 healthy controls, 48 fecal samples collected during hospitalization and 13 fecal samples collected during a 1-month follow-up [25] and a cohort of data obtained in Shanghai during early waves of the COVID-19 pandemic that comprised samples from 8 healthy controls and 62 fecal samples from COVID-19 patients collected during hospitalization [19]. Then, we collected paired oral, fecal, and serum samples from 983 antibiotic treatment-naïve mild COVID-19 patients during hospitalization (Table 1). Paired oral, fecal, and serum samples from 25 normal controls were collected simultaneously. After 3 months of follow-up, we identified 45 patients with GI symptoms related to long COVID-19. Details regarding these patients and normal controls are shown in Table 2, and details of the disease occurrence in enrolled patients with GI symptoms of long COVID-19 at the time of the follow-up are shown in Table 3. The overall morbidity of GI-related long COVID-19 in this cohort was approximately 4.6% (45/983). Moreover, we also collected 8 paired oral and fecal samples from patients without GI symptoms after the 3-month follow-up, and the details of these patients are shown in Table 4.

### Diversity of gut microbiota composition in long COVID-19 patients and patients who recovered from mild COVID-19

**Fig. 2:**
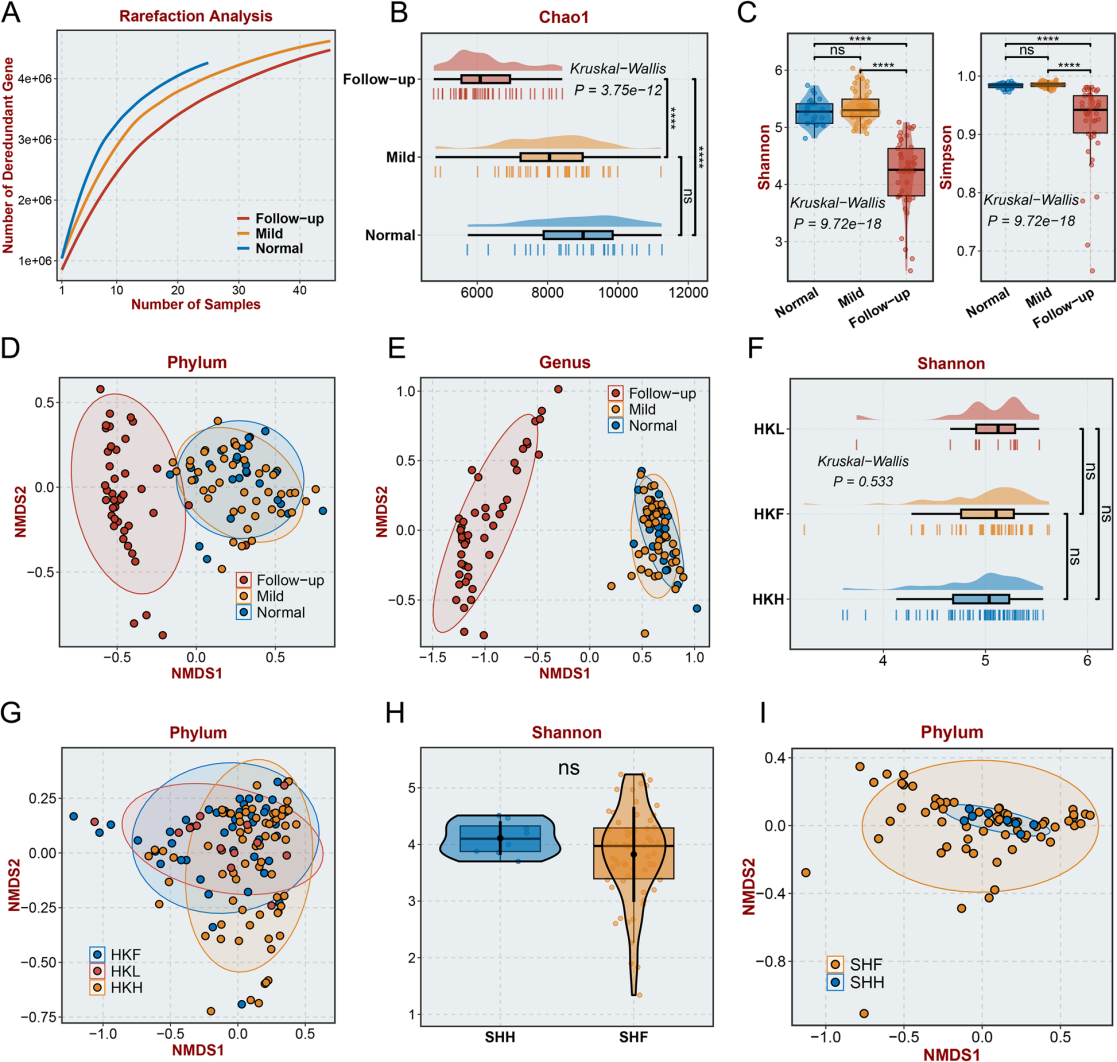
Diversity differences in the gut microbiome.** A** Rarefaction analysis showed that as the number of fecal samples increased, the number of nonredundant genes approached saturation in the mild (*n* = 45), follow-up (*n* = 45), and normal (*n* = 25) groups. **B**-**D** The Chao1 (**B**), Shannon (**C**), and Simpson (**D**) indices of the gut microbiome. **E**,**F** The NMDS based on the relevant abundance of the microbiome at the phylum level (**E**) and genus level (**F**). **G** The Shannon index of the gut microbiome in the Hong Kong cohort. “ns” represents no significance. **H** Phylum-level NMDS analysis of the gut microbiome in the Hong Kong cohort. **I** The Chao1 and Shannon index of the gut microbiome in the Shanghai cohort. **J** Phylum-level NMDS analysis of gut microbiota in the Shanghai cohort. The mild group consisted of samples obtained from patients diagnosed with mild COVID-19; the follow-up group consisted of samples obtained from patients at the 3-month follow-up after discharge; and the normal group consisted of normal samples. HKL consisted of samples from follow-up mild COVID-19 patients in the Hong Kong cohort after discharge. HKF consisted of samples obtained from patients diagnosed with mild COVID-19. HKH consisted of normal samples. SHL consisted of samples from mild COVID-19 patients. SHH consisted of normal samples. “ns” represents no significance, “ns” represents no significance, *p < 0.05, **p < 0.01, ***p < 0.001, and ****p < 0.0001 (Student’s *t* test)

To investigate the differences between the gut microbiota of long COVID-19 patients with mild disease when assessed during hospitalization or follow-up and the gut microbiota of healthy controls, we performed metagenomic sequencing using 25 fecal samples from healthy controls and 45 paired fecal samples collected from long COVID-19 patients during hospitalization and at the 3-month follow-up. As the number of samples increased, the number of nonredundant genes approached saturation in each group (Fig. 2A). The alpha diversity calculated in the follow-up group was significantly lower than that in the mild group and normal group according to the Chao1 index (Fig. 2B, *P* < 0.0001), Shannon index (Fig. 2C, *P* < 0.0001), Simpson index (Fig. 2D, *P* < 0.0001), and ACE index (Additional file 3: Figure S1A, *P* < 0.0001), illustrating that the diversity of the gut microbiota significantly decreased in the follow-up group. The beta diversity also exhibited significant alterations in the follow-up group based on NMDS (Non-metric Multidimensional Scaling) analysis of bacterial abundance at the phylum level (Fig. 2E), genus level (Fig. 2F), and species level (Additional file 3: Figure S1B), indicating that there were potential differences in the gut microbiota between the follow-up group and the other two groups. Additionally, PCoA(Principal Coordinates Analysis) and PCA (Principal Component Analysis) at the phylum level (PCoA: Figure S1C, PCA: Figure S1F), genus level (PCoA: Additional file 3: Figure S1D, PCA: Additional file 3: Figure S1G), and species level (PCoA: Additional file 3: Figure S1E, PCA: Additional file 3: Figure S1H) confirmed our findings of significant alterations in beta diversity. Moreover, our results revealed no significant differences in the gut microbiota between the mild group and the normal group (Fig. 2C–F, no significance). To confirm our findings and investigate whether there were significant differences in the gut microbiota between mild COVID-19 patients during hospitalization or follow-up and healthy controls, two previously published datasets from studies that investigated gut microbiota changes after COVID-19 were downloaded and analyzed, including a cohort from Hong Kong [25] and a cohort from Shanghai [19]. The alpha and beta diversity of the gut microbiota showed no significant differences among the follow-up group, mild group, and normal group in the Hong Kong cohort based on the Shannon index (Hong Kong cohort: Fig. 2G, no significance, Shanghai cohort: Fig. 2I, no significance), Chao1 index (Hong Kong cohort: Additional file 3: Figure S1I, no significance), and analysis of NMDS2 at the phylum level (Hong Kong cohort: Fig. 1H, Shanghai cohort: Fig. 1J) and genus level (Hong Kong cohort: Additional file 3: Figure S1J, Shanghai cohort: Additional file 3: Figure S1H). Additionally, we also collected 8 fecal samples from patients without GI symptoms after the 3-month follow-up and sequenced these samples and fecal samples from these patients during hospitalization. The alpha diversity of the gut microbiota showed no significant differences between the mild group and follow-up group, based on the ACE, Chao1, Shannon, and Simpson index (Additional file 3: Figure S2A, no significance). The beta diversity of the gut microbiota also showed no significant differences according PCoA (Additional file 3: Figure S2B), NMDS (Additional file 3: Figure S2C), and PCA analysis (Additional file 3: Figure S2D).

### Variations in the gut microbiome in long COVID-19 patients during hospitalization and at follow-up

**Fig. 3:**
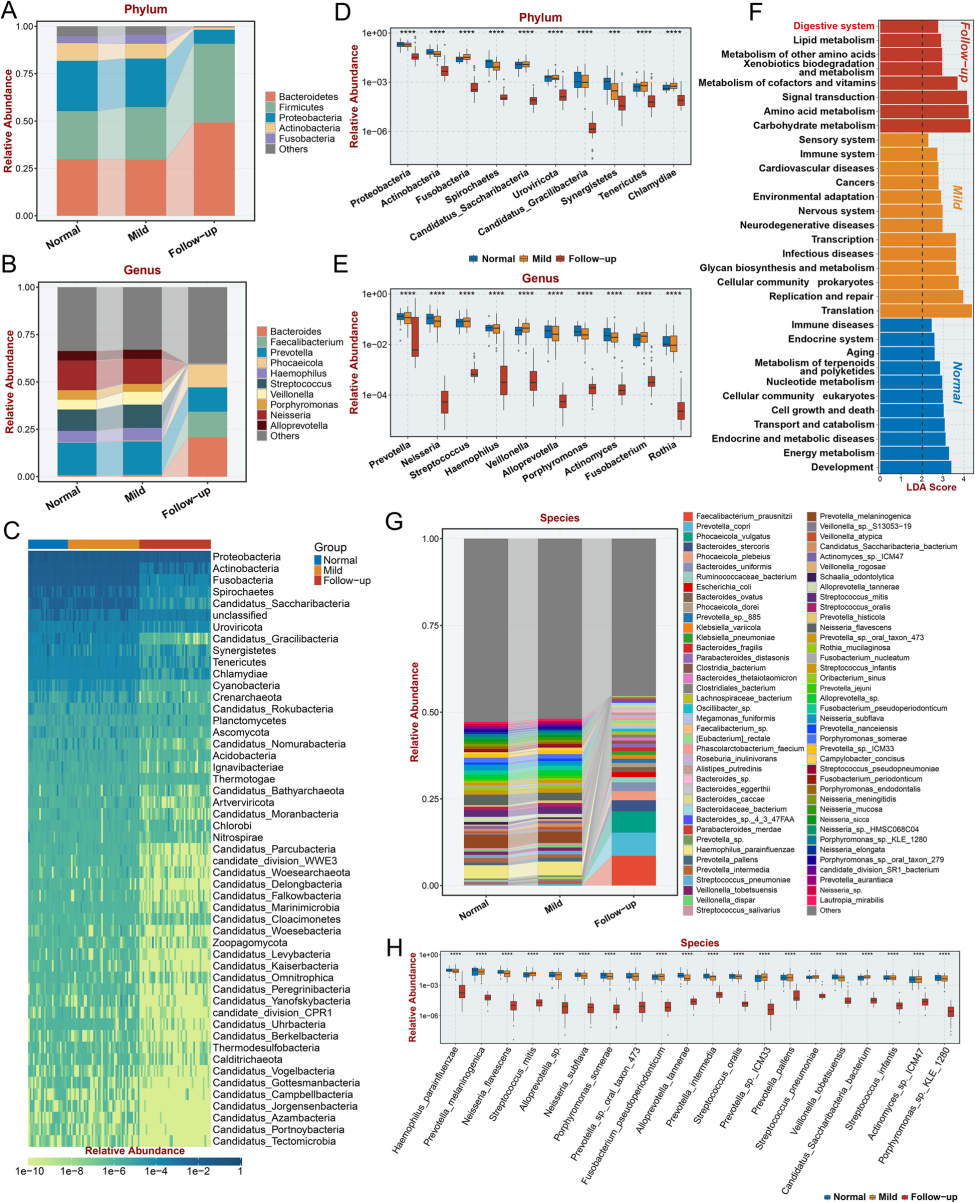
Specific microbiomes with significantly reduced relative abundance in follow-up patient fecal samples. **A**,**B** Average relative abundance of the top five phyla (**A**) and top 10 microbial genera (**B**) detected in feces from normal individuals, in-hospital patients, and their follow-up within 3 months after discharge. **C** Microbial phyla with significantly lower relative abundance in fecal samples from 3-month follow-up patients compared with normal samples or in-hospital patients. **D**,**E** The top 10 microbial phyla (**D**) and genera (**E**) with significantly lower relative abundance in fecal samples from 3-month follow-up patients compared with normal samples or in-hospital patients. **F** Significantly associated Kyoto Encyclopedia of Genes and Genomes (KEGG) functions of the follow-up, mild, and normal groups, with an LDA (linear discriminant analysis) score > 1. **G** Average relative abundance of the top 79 microbial species detected in fecal samples from the normal, mild, and follow-up groups. **H** The top 20 microbial species with significantly lower relative abundance in fecal samples of follow-up patients compared with normal samples or mild patients. “ns” represents no significance, *p < 0.05, **p < 0.01, ***p < 0.001, and ****p < 0.0001 (Student’s *t* test)

To identify the specific gut microbes that were differentially abundant between the follow-up group and the mild group or normal group, the differences in the abundance of the top 5 phyla (Fig. 3A) and top 10 genera (Fig. 3B) among groups were evaluated. The abundances of *Prevotella*, *Haemophilus*, *Streptococcus*, *Veillonella*, *Porphyromonas*, *Neisseria*, and *Alloprevotella* were decreased and almost absent in the follow-up group (Fig. 3B). Since we discovered that both the alpha and beta diversity of the gut microbiome were significantly decreased in the follow-up group, we next identified which gut microbes had significantly decreased abundance in the follow-up group at the phylum level; these included *Proteobacteria*, *Actinobacteria*, *Fusobacteria*, *Spirochaetes*, and *Uroviricota* and some other bacterial phyla with low abundance (Fig. 3C). The top 10 differentially abundant bacteria were identified at the phylum (Fig. 3D, *P* < 0.001) and genus levels (Fig. 3E, *P* < 0.0001). These bacteria all had decreased abundance in the follow-up group, suggesting that decreased bacterial abundance is a remarkable characteristic in the lower alimentary tract of patients with GI-related long COVID-19. Moreover, we investigated the enriched pathways among the differentially enriched bacteria in each group (Fig. 3F, *P* < 0.05) and identified the differentially enriched bacteria at the species level (Fig. 3H, *P* < 0.0001). The top 79 average relative abundance of the microbial species detected in fecal samples from the normal, mild, and follow-up groups was also visualized in Fig. 3G. Interestingly, the “Digestive system” KEGG (Kyoto Encyclopedia of Genes and Genomes) term was significantly enriched in the follow-up group, suggesting a potential impact of differentially enriched bacteria in the GI tract on patients with GI symptoms of long COVID-19 (Fig. 3F, *P* < 0.05). Multiple metabolism terms, including “Lipid metabolism,” “Metabolism of other amino acids,” “Metabolism of cofactors and vitamins,” “Amino acid metabolism,” and “Carbohydrate metabolism” were also enriched in the gut microbiome of follow-up group, indicating metabolic disorder plays a pivotal role in GI symptoms of long COVID-19. Moreover, “immune system” and “infectious disease” was enriched in mild COVID-19 group, emphasized the dysfunction of immune system in mild COVID-19 is associated the alteration of gut microbiome.

### Variations in the oral microbiome in long COVID-19 patients during hospitalization and at follow-up

**Fig. 4:**
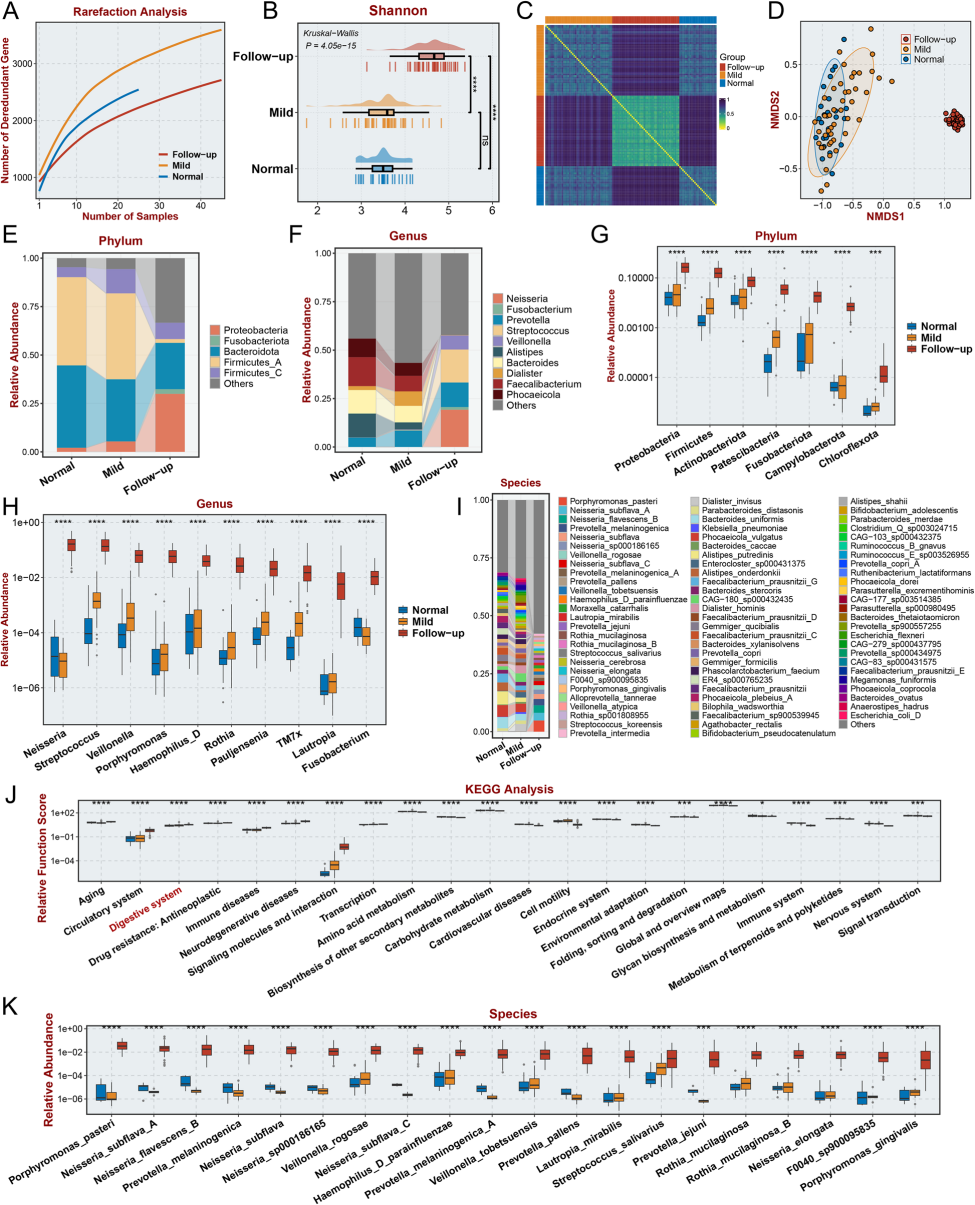
Diversity differences in the oral microbiome and specific microbiomes with significantly increased relative abundance in follow-up patient saliva samples. **A** Rarefaction analysis showed that as the number of saliva samples increased, the number of nonredundant genes approached saturation in the mild (*n* = 45), follow-up (*n* = 45), and normal (*n* = 25) groups. **B** The Shannon index of the oral microbiome in the follow-up group. **C** The Jaccard distance between the different groups reflects the low similarity of microbial composition in saliva samples from the follow-up group to the normal group and mild COVID-19 patients. **D** The NMDS based on the relevant abundance of the microbiome in the follow-up group, normal, and mild groups. **E**,**F** Average relative abundance of the top five phyla (**E**) and top 10 microbial genera (**F**) detected in saliva from normal individuals, in-hospital patients, and their follow-up within 3 months after discharge. **G**,**H** The microbial phyla (**G**) and top 10 genera (**H**) with significantly higher relative abundance in fecal samples from 3-month follow-up patients compared with normal samples or in-hospital patients **I**. Average relative abundance of top 79 microbial species detected in oral microbiota from the Normal, Mild, and Follow-up samples. **J** The KEGG functions that were significantly enhanced or reduced in the follow-up group. **K** The top 20 microbial species with significantly high relative abundance in saliva samples of follow-up patients compared to normal samples or mild patients. “ns” represents no significance, *p < 0.05, ** *p* < 0.01, ##*p* < 0.01, ***p < 0.001, and ****p < 0.0001 (Student’s *t* test)

Next, we performed 2bRAD-M analysis using 25 saliva samples collected from healthy controls and 45 paired saliva samples from long COVID-19 patients collected during hospitalization and at the 3-month follow-up. The rarefaction curve indicated that the sample size used in this study was appropriate for the following analysis (Fig. 4A). The alpha diversity in the follow-up group was significantly higher than that in the mild group and normal group according to the Shannon index (Fig. 4B, *P* < 0.0001), Chao1 index (Additional file 3: Figure S3A), and Simpson index (Additional file 3: Figure S3B). The similarity of the composition of the oral microbiome was high in the follow-up group based on visualization of the Jaccard distance (Fig. 4C), suggesting that patients with GI symptoms of long COVID-19 may have consistent changes in the oral microbiome. The beta diversity was also significantly altered in the follow-up group based on NMDS2 analysis (Fig. 4D), PCoA (Additional file 3: Figure S3C), and PCA of bacterial abundance (Additional file 3: Figure S3D). To investigate whether there were significant differences in the oral microbiota of patients without GI symptoms between during hospitalization or follow-up, we also collected 8 saliva samples from patients without GI symptoms after the 3-month follow-up and sequenced these samples and saliva samples from these patients during hospitalization. The alpha diversity of the oral microbiota showed no significant differences between the mild group and follow-up group, based on the Chao1, Shannon, and Simpson index (Additional file 3: Figure S4A, no significance). The beta diversity of the oral microbiota also showed no significant differences according PCoA (Additional file 3: Figure S4B), NMDS (Additional file 3: Figure S4C), and PCA (Additional file 3: Figure S4D) analysis. To identify the specific oral microbes that were differentially abundant between the follow-up group and the mild group or normal group, the top 5 phyla (Fig. 4E) and top 10 genera (Fig. 4F) with differences in abundance among groups were identified. Moreover, 7 bacteria with significantly increased abundance were observed at the phylum level (Fig. 4G, *P* < 0.001), and the top 10 significantly altered bacteria at the genus level (Fig. 4H, *P* < 0.0001) were identified (sorted by the relative abundance in the follow-up group). These bacteria all had increased abundance in the follow-up group, suggesting that increased bacterial abundance is a remarkable characteristic of the oral cavity of patients with GI symptoms of long COVID-19. Moreover, we identified the differentially enriched bacteria at the species level (Fig. 4I, K, *P* < 0.001) and the enriched pathways among the differentially enriched bacteria in each group (Fig. 4J, *P* < 0.05). Intriguingly, the “Digestive system” KEGG term was also significantly enriched (Fig. 4J, *P* < 0.05). Some metabolic pathways, including “Amino acid metabolism,” “Biosynthesis of other secondary metabolites,” and “Glycan biosynthesis and metabolism” were enriched in the oral microbiome of follow-up group, consistent with the results acquired from analysis of gut microbiome in follow-up group. These results indicate the significance of change metabolites resulting from altered bacteria both upper and lower digestive tract in GI symptoms related long COVID-19.

### Colonization of the oral cavity by gut microbes may occur in patients with GI symptoms of long COVID-19

**Fig. 5:**
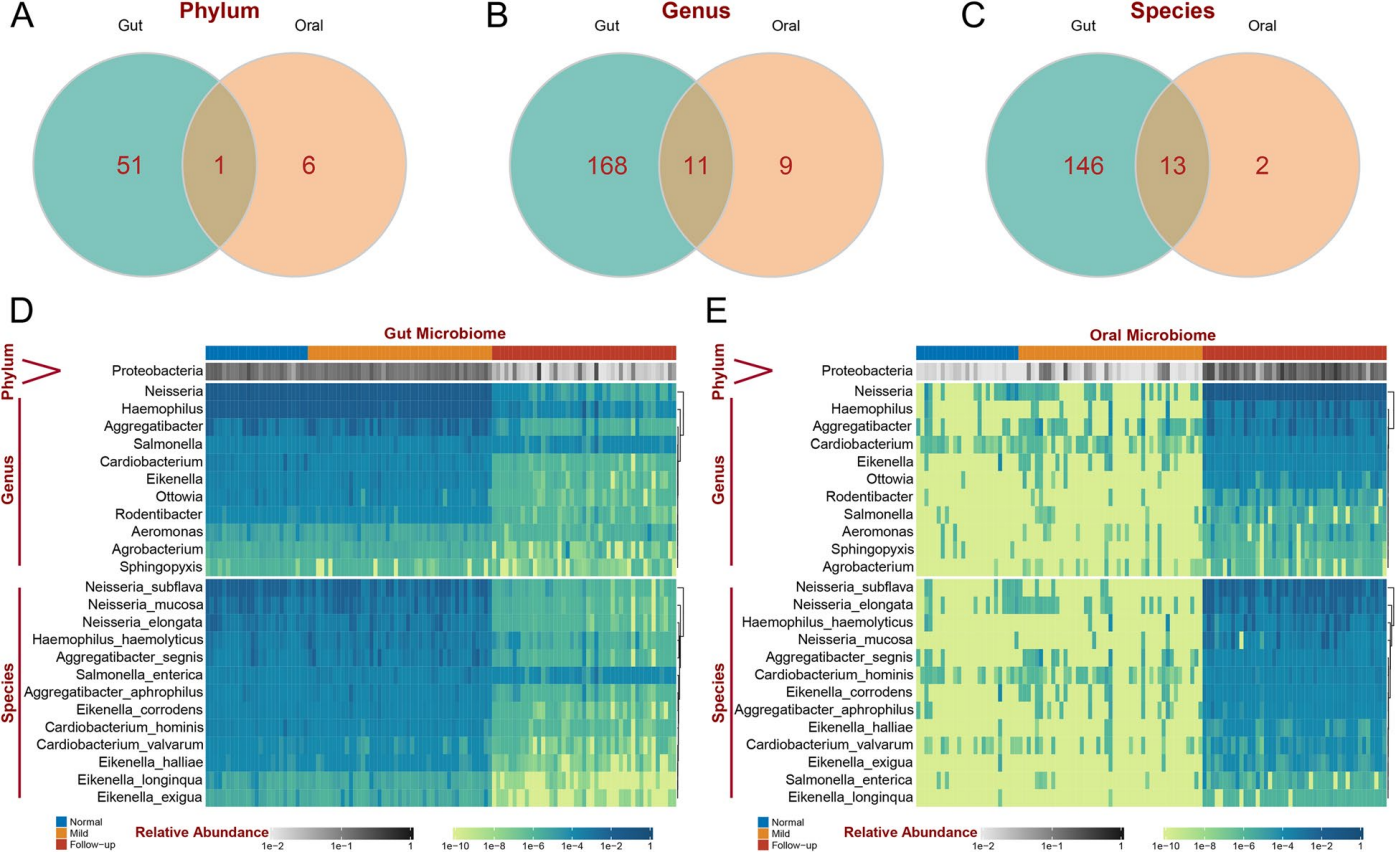
The intersection of specific differential oral and gut microbiomes. **A**–**C** Venn diagram displaying the number of gut-specific differential microbiomes intersected with mouth-specific differential microbiomes at the phylum (**A**),genus (**B**), and species (**C**) levels. **D**,**E** The heatmap displays the relevant abundance of intersecting microbiomes in gut samples (**D**) and oral samples (**E**)

A previous study has proven that enteric microorganisms can be transmitted by fecal–oral routes through direct contact or indirect exposure to contaminated fluids and foods [53]. Hence, we focused on the differential bacteria that were common between saliva samples and fecal samples. Proteobacteria was the only common differentially abundant bacteria at the phylum level (Fig. 5A), 11 common differentially abundant genera of *Proteobacteria* were identified, including *Neisseria*, *Haemophilus*, *Aggregatibacter*, *Cardiobacterium*, *Eikenella*, *Ottowia*, *Rodentibacter*, *Salmonella*, *Aeromonas*, *Sphingopyxis*, and *Agrobacterium*(Fig. 5B). Thirteen common differentially abundant species of Proteobacteria were identified, including *Neisseria_subflava*, *Neisseria_elongata*, *Haemophilus_haemolyticus*, *Neisseria_mucosa*, *Aggregatibacter_segnis*, *Cardiobacterium_hominis*, *Eikenella_corrodens*, *Aggregatibacter_aphrophilus*, *Eikenella_halliae*, *Cardiobacterium_valvarum*, *Eikenella_exigua*, *Salmonella_enterica*, and *Eikenella_longinqua*. The relative abundances of these bacterial genera in fecal samples and saliva samples are visualized in Fig. 5D and E, respectively. *Neisseria* was the most enriched bacteria in both metagenome data obtained from fecal samples and 2bRAD-M data obtained from saliva samples. Additionally, most of the bacteria showed little difference between the healthy control group and the COVID-19 group. These results indicate that the different kinds of common bacteria exhibit opposite trends in their levels in the upper and lower alimentary tract, and these bacteria include certain genus and species in *Proteobacteria*, suggesting the possibility of ectopic bacteria in the upper and lower digestive tract. To identify whether the alteration of these bacteria is unique in long COVID-19 patients with GI symptoms, we investigate the expression of these bacteria in COVID-19 rehabilitee without GI symptoms after 3 months follow-up. The results shows there is no significant changes among these bacteria both in phylum (Additional file 3: Figure S2E), genus (Additional file 3: Figure S2F), and species level (Additional file 3: Figure S2G) of fecal microbiota and in phylum (Additional file 3: Figure S4E), genus (Additional file 3: Figure S4F), and species level (Additional file 3: Figure S4G) of oral microbiota.

### Differences in serum metabolite levels exist in patients with GI symptoms of long COVID-19 and are correlated with levels of ectopic bacteria

**Fig. 6:**
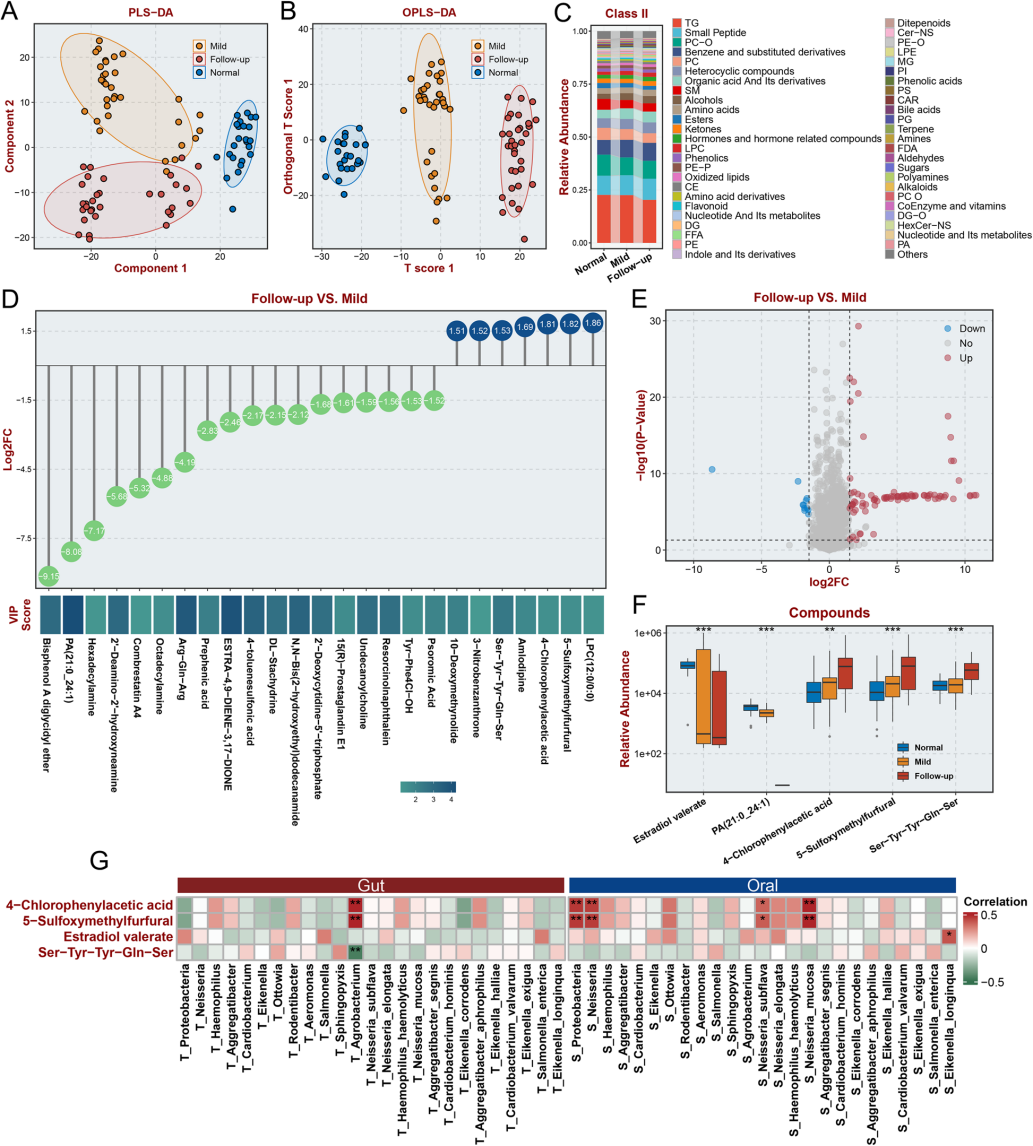
Alteration of specific metabolites in patient serum and association between altered metabolites and microbial changes. **A**,**B** The PLS-DA (**A**) and OPLS-DA (**B**) analyses suggest differences in metabolites of serum samples from different groups. **C** Average relative abundance of the top 49 Class II metabolites in serum from normal individuals, in-hospital patients, and their follow-up within 3 months after discharge. **D** The differential abundance of serum Class II metabolites (Log2 FC > 1.5 or <  − 1.5, VIP > 1.0, *p* < 0.05) in the follow-up and mild patient groups is presented as a lollipop chart. **E** Annotated Class II metabolites were analyzed and compared between the follow-up and normal groups (log2 FC > 1.5 or <  − 1.5, VIP > 1.0, *p* < 0.05). **F** Up- and downregulated Class II metabolites in the follow-up group compared with the normal group or the mild COVID-19 group. **G** Association analysis between the abundance of significantly altered Class II metabolites in serum and altered abundance of digestive tract microbiomes at the phylum, genus, and species levels in the follow-up group. “ns” represents no significance, *p < 0.05, ** *p* < 0.01, ***p < 0.001, and ****p < 0.0001 (Student’s *t* test)

A previous study illustrated that alterations in the microbiota in the GI tract could affect serum metabolic status [54]. The imbalance of serum metabolism is a key characteristic of COVID-19 patients [55, 56]. Thus, we aimed to identify the differential metabolites between long COVID-19 patients with GI symptoms and healthy controls or hospitalized patients through UPLC‒MS/MS-based widely targeted metabolomics. PLS-DA analysis showed significant differences among the three groups of samples (Fig. 6A). OPLS-DA indicated that samples from the follow-up group showed greater variation from samples collected from the mild group than samples collected from the normal group, suggesting that metabolite differences were sustained and tended to increase in the follow-up group (Fig. 6B). The changes in the levels of the top 50 secondary metabolites with the highest relative abundance among the three groups are shown in Fig. 6C. Additionally, the variation in the levels of the top 27 primary metabolites and top 49 compounds with the highest relative abundance among the three groups are shown in Additional file 3: Figure S5A and Additional file 3: Figure S5B, respectively. Then, we identified differential metabolites among the three groups through pairwise comparison. Most of the differential metabolites had lower levels in the follow-up group than in the mild group (Fig. 6D, Additional file 4: Table S2), and most of the differential metabolic compounds had higher levels in the follow-up group than in the normal group (Fig. 6E, Additional file 5: Table S3), suggesting that the altered metabolite levels in patients with GI symptoms of long COVID-19 had partly recovered. Then, 5 differential metabolites were identified by evaluating the respective intersections of metabolites with increased levels and metabolites with decreased levels between these two comparisons (Fig. 6F). Then, we performed Pearson correlation analysis to assess the correlation between the levels of these differential metabolites and the expression of the differential gut and oral microbes identified during the previous experiment at the genus level (Fig. 6G). The levels of oral *Neisseria* had a strong correlation with the levels of 4-chlorophenylacetic acid and 5-sulfoxymethylfurfural. The levels of oral *Lautropia* were strongly related to the levels of 4-chlorophenylacetic acid, 5-sulfoxymethylfurfural, and estradiol valerate. These results indicated that there were important oral and gut bacteria involved in the progression of the metabolic disorders that occurred in patients with GI symptoms of long COVID-19. Moreover, we explored the correlation between the levels of these differential metabolites and the expression of enriched differential gut and oral microbes identified during the previous experiment at the species level (Additional file 3: Figure S5C). Eikenella longinqua levels in the gut were identified to have a high positive correlation with TG (triglyceride) levels, and those in the oral microbiota had a negative correlation (*P* < 0.05).

## Discussion

A previous long COVID-19 study revealed that alterations in the microbiota exist in COVID-19 patients, including changes in *Eubacterium*, *Faecalibacterium*, *Coprobacillus*, *Clostridium ramosum*, *Roseburia*, and *Bifidobacterium* in the gut [5, 6, 57] and *Leptotrichia*, *Streptococcus*, and *Actinobacillus* in the oral cavity [58]*.* Additionally, alterations in the microbiota have been identified in long COVID-19 patients, including changes in *Ruminococcus gnavus*, *Bacteroides vulgatus*, and *Faecalibacterium prausnitzii* in the gut [28] and *Leptotrichia*, *Prevotella*, and *Fusobacterium* in the oral cavity. However, these studies were based on the analysis of primitive COVID-19 cases, including a large number of severe cases; the results exhibit nonnegligible bias and cannot be used to characterize the current pandemic. The most important source of bias originates from the fact that the virus involved in the current pandemic is Omicron, a variant of SARS-CoV-2 that is highly infectious and associated with a low incidence of severe cases [36]. Additionally, some reports have indicated that a considerable proportion of patients who recovered from mild COVID-19, which ranges between 10 and 35%, experienced persistent symptoms [37]. However, studies focusing on these biases have been rare until recently. In our current study, we investigated the changes in the oral and fecal microbiomes with serum alterations in COVID-19 patients and recovered patients with GI-related long COVID-19 compared to healthy controls, and we conclude with a novel understanding of this process.

The bioinformatic analyses conducted in our study revealed that the abundance of microbes in the gut was similar between the healthy control group and mild patients when evaluated during hospitalization or at follow-up, suggesting that there are no substantial differences in the gut microbiome between mild COVID-19 patients and recovered patients. Some studies have illustrated that the abundance of gut microbes in COVID-19 patients is significantly different from that in healthy controls [5, 6], even several months after discharge [29]. The results from previous studies are not consistent with our conclusion due to the mild severity of COVID-19 in the patients enrolled in our bioinformatic study, which used data collected from previous studies. Our current results illustrate that the changes that occur in bacteria are minor in mild COVID-19 patients without long COVID-19 syndrome when compared at the time of hospitalization and at follow-up.

The most important aspect of our study was the characterization of the oral and gut microbiota in patients with GI-related long COVID-19. First, in our current cohort, which was obtained during the second wave of the pandemic in Shanghai from April to June 2022, among the 983 enrolled patients, 45 patients were identified as having GI symptoms related to long COVID-19 (4.6%). A previous study reported that 44–66% of patients had GI symptoms related to long COVID-19 [59, 60]. The high rate of sequelae observed in the previous study could have been because most of the patients enrolled in this study were moderate and severe COVID-19 patients. Another recent study from India enrolled 320 patients, including 74.3% with mild COVID-19 and 8.4% of COVID-19 patients with functional GI disorders 3 months after discharge [61]. This result is similar to our findings, suggesting that an incidence of GI-related long COVID-19 of approximately 5% exists in the present pandemic.

Second, the comparison of metagenome data obtained from fecal samples between patients during hospitalization or at follow-up and healthy controls revealed that α-diversity had a decreasing trend and that there was a significant discrepancy in β-diversity in patients with GI long COVID-19 symptoms. A previous study reported that the α-diversity of the gut microbiota in long COVID-19 patients was significantly lower than that in healthy controls or discharged patients without long COVID-19 [28]. Our study supports this point of view. However, most of the different bacteria at the genus level identified in our study are different from those identified in previous studies. The differences in the diets between the enrolled patients may have contributed to this heterogeneity in the gut microbiota. In fact, long COVID-19-related studies from different cities and countries conducted during the early wave of the pandemic have also shown a large difference in gut microbiota [27, 28].

Regarding the changes in the oral microbiota, no significant difference was observed in α-diversity and β-diversity between hospitalized mild COVID-19 patients and healthy controls. Previous studies reported that the α-diversity of the oral microbiota was significantly decreased and that the β-diversity of the oral microbiota was significantly changed during hospitalization in COVID-19 patients [31, 58]. However, these studies had a large proportion of moderate and severe COVID-19 patients, which could explain the discrepancy between these studies and our current results. Additionally, a trend for increased α-diversity and a significant difference in β-diversity were observed between patients with GI symptoms related to long COVID-19 during hospitalization or at follow-up and healthy controls based on 2bRAD-M analysis of saliva samples. To the best of our knowledge, this is the first study to identify the characteristics of the oral microbiota in patients with GI symptoms related to long COVID-19, which could enhance the value of our study.

In previous studies, specific differences in the metabolomic profile of serum between mild COVID-19 patients and healthy controls have been identified [55, 56, 62]. However, the changes in serum metabolic profiles that occur in patients with GI-related long COVID-19 have yet to be elucidated. Our study revealed the profile of differential serum metabolites in patients with GI-related long COVID-19 and identified differential metabolites that were consistently different between the mild COVID-19 group vs. the normal group and the follow-up group vs. the normal group, including two metabolites with lower levels in the follow-up group, which included estradiol valerate and PA, and three metabolites with higher levels in the follow-up group, including 4-chlorophenylacetic acid, 5-sulfoxymethylfurfural, and Ser-Tyr-Cln-Ser. Estradiol valerate is a kind of estrogen and has lower levels in patients with GI-related long COVID-19 than in healthy controls and in hospitalized patients. Estrogen regulates the metabolism of specific compounds and growth and development, and it has several target tissues, including the reproductive system, cardiovascular system, digestive system, and bones [63]. Some studies have reported on the protective role of estrogen supplementation in COVID-19 treatment based on clinical [64] and basic research [65]. Indeed, studies of other patient cohorts worldwide confirmed the presence of male predisposition to higher morbidity and mortality [66–68]. Our study further indicates that a lower level of serum estrogen widely exists in patients with GI-related long COVID-19. Moreover, 5-sulfoxymethylfurfural is a carcinogen with detrimental effects (mutagenic, genotoxic, organotoxic, and enzyme inhibitory) [69]. Further studies are needed to investigate the potential role of these two metabolites in the process of GI-related long COVID-19.

Moreover, a previous study revealed that the abnormal ectopic colonization of the upper and lower alimentary tracts by specific bacteria could promote some GI diseases [39, 53, 70]. However, the dynamic changes in the microbiota that occur in the oral cavity and intestine have yet to be elucidated. In our current study, *Neisseria* had a lower abundance in the colon and a higher abundance in the oral cavity of patients with GI-related long COVID-19, and the abundance of oral *Neisseria* was highly correlated with the levels of differential serum metabolites. A previous study reported that the abundance of the *Neisseria* genus was elevated in the oral microbiota of COVID-19 patients, and they also reported that white blood cell and lymphocyte counts positively correlated with the abundance of the *Neisseria* genus [8]. According to these findings, we hypothesize that the ectopic colonization of the oral cavity by a series of bacteria in the *Proteobacteria* phylum originating from the colon and led by *Neisseria* is a risk factor for GI-related long COVID-19. This concept emphasizes the importance of maintaining personal hygiene, especially hand hygiene, after COVID-19 patients are discharged. Indeed, further research is needed to verify this concept.

However, this study contains some limitations. First, our follow-up investigation was based on telephone, which may not comprehensively reflect patient status. To overcome this limitation, we will continue to perform follow-up on these enrolled patients in the clinic. Second, the concept of abnormal ectopic colonization of the upper and lower alimentary tracts with specific bacteria needs to be further validated. To overcome this limitation, we will collect colon tissue and oral tissue from enrolled patients for further validation.

## Conclusions

In summary, first, our study revealed that the oral and gut microbiomes of mild COVID-19 patients without long COVID-19 when evaluated at follow-up displayed little difference from those evaluated during hospitalization or in healthy controls. Second, our study emphasizes that mild COVID-19 patients with GI-related long COVID-19 exhibit ectopic colonization of the oral microbiome by gut microbes and a disturbance in serum metabolites.

At present, the COVID-19 pandemic has entered its fourth year, the virulence of SARS-COV-2 has become less lethal as the virus has spread, and millions of people have become infected and have experienced mild COVID-19. First, according to our results, we propose a potential pathogenic mechanism for the occurrence of GI-related long COVID-19, whereby ectopic colonization of the oral cavity by some abundant genera in the Proteobacteria phylum from the gut leads to GI-related long COVID-19. Because this potential susceptibility for COVID-19 patients to ectopic colonization after recovery was observed, our study emphasizes the importance of adhering to hand hygiene after recovery from COVID-19.

Second, our findings promote the use of microbial treatment and metabolite therapy for mild COVID-19 patients diagnosed with long COVID-19 syndrome after discharge. Moreover, as the strong relevance between the ectopically colonized bacteria and some potential harmful metabolites in serum was identified in our study, further research is needed to explore the mechanism of some abundant genera in the Proteobacteria phylum to GI-related long COVID-19 through changes in serum metabolites.

## Supplementary Information

**Additional file 1. **Supplementary materials and methods: The details of metagenomic bioinformatic analysis and 2bRAD-M bioinformatic analysis.

**Additional file 2: Table S1.** Clinical characteristics of the enrolled patients for serum UPLC-MS/MS-based widely targeted metabolomics.

**Additional file 3: Figure S1.** Diversity differences in the gut microbiome. **Figure S2.** Comparison of gut microbiota between recovers without GI symptoms at 3 months of follow-up after COVID-19 and those with mild disease. **Figure S3.** Diversity differences in the oral microbiome. **Figure S4.** Comparison of oral microbiota between recovers without GI symptoms at 3 months of follow-up after COVID-19 and those with mild disease. **Figure S5.** Alteration of specific metabolites in patient serum.

**Additional file 4:** **Table S2.** Differential metabolites in serum between follow-up group and mild group.

**Additional file 5: Table S3.** Differential metabolites in serum between follow-up group and normal group.

## Data Availability

The metagenomics sequencing dataset was deposited in the National Center for Biotechnology Information Sequence Read Archive under BioProject accession number PRJNA908301. The previous Metagenomic data from Shanghai were obtained from the NODE (https://www.biosino.org/node/project/detail/OEP002590) [71]. The Shanghai cohort [19] dataset included data from 62 patients with mild COVID-19 and 8 healthy controls. Another datasets from SRA (https://www.ncbi.nlm.nih.gov/bioproject/PRJNA689961) databases [72] was obtained as the Hong Kong cohort, including data from 48 patients with mild disease, 13 follow-up patients, and 70 healthy controls [25].

## Abbreviations

2bRAD-M: 2BRAD sequencing for microbiome
ACE2: Angiotensin-converting enzyme 2
COVID-19: Coronavirus disease 2019
GI: Gastrointestinal
KEGG: Kyoto Encyclopedia of Genes and Genomes
LDA: Linear discriminant analysis
LEfSe: Linear discriminant analysis effect size
NMDS2: Non-metric Multidimensional Scaling 2
OPLS-DA: Orthogonal Partial Least Squares-Discriminant Analysis
OSC: Orthogonal signal correction
PCA: Principal Component Analysis
PCoA: Principal Coordinates Analysis
PLS-DA: Partial least squares-discriminant analysis
VIP: Variable importance in projection
