# Supplementary material for: Gastrointestinal symptoms of long COVID-19 related to the ectopic colonization of specific bacteria that move between the upper and lower alimentary tract and alterations in serum metabolites
Source: BMC Med. 2023 Jul 19;21:264. doi: 10.1186/s12916-023-02972-x (PMC10355065; doi:10.1186/s12916-023-02972-x)
Supplement: Supplementary file 1 — Additional file 1. Supplementary materials and methods: The details of metagenomic bioinformatic analysis and 2bRAD-M bioinformatic analysis. [file 12916_2023_2972_MOESM1_ESM.docx]

**Supplementary materials and methods**

**Metagenomic bioinformatic analysis**

The libraries were sequenced on an Illumina NovaSeq 6000 platform, and 150 bp paired-end reads were generated. Sequences in the FastQ file were trimmed and filtered using Trimmomatic (v 0.36) (43). Host pollution control was needed. The postfiltered paired-end reads were aligned against the host genome using Bowtie2 (v 2.2.9), and the unaligned reads were discarded. Metagenome assembly was performed using MEGAHIT (v 1.1.2) after obtaining valid reads (44,45). Gaps inside the scaffold were used as a breakpoint to interrupt the scaffold into new contigs (Scaftig), and new scaftigs with lengths > 500 bp were retained. Open reading frame (ORF) prediction of assembled scaffolds was performed using prodigal (v 2.6.3)(46), and the results were translated into amino acid sequences. The nonredundant gene sets were built for all predicted genes using CDHIT (v 4.5.7). The clustering parameters were 95% identity and 90% coverage. The longest gene was selected as the representative sequence of each gene set. Clean reads of each sample were aligned against the nonredundant gene set (95% identity) using Bowtie2 (v 2.2.9), and the abundant information of the gene in the corresponding sample was counted.

The taxonomy of the species was obtained using the corresponding taxonomy database of the NR Library, and the abundance of the species was calculated using the corresponding abundance of the genes. To generate the abundance profile at the corresponding taxonomic level, abundance statistics were performed at each level, including domain, kingdom, phylum, class, order, family, genus, and species. The gene set representative sequence (amino acid sequence) was annotated with the NR, KEGG (47,48), COG (49), SWISSPROT, and GO databases with an e-value of 1e-5 using DIAMOND (v 0.9.7) (50). The gene sets were compared with the CAZy database using the corresponding tool hmmscan (v 3.1) to obtain information regarding the carbohydrate active enzyme corresponding to the gene, and the carbohydrate activity was then calculated using the sum of the gene abundances corresponding to the carbohydrate active enzyme abundance (51).

The principal component analysis (PCA) and plotting of the taxonomy abundance spectrum or functional abundance spectrum were carried out using R software (4.1.3), and the results of the equidistant matrix of PCoA and Non-metric Multidimensional Scaling (NMDS) were calculated and analyzed. The differences between the mild group and the follow-up group were evaluated by paired T test or Wilcoxon test, while the differences between the mild group and normal group were evaluated by t test or Wilcoxon test. Additionally, differences between multiple groups were evaluated by the Kruskal‒Wallis test.

**Identification of species-specific 2bRAD-M markers from the most comprehensive genomic database**

First, a total of 173,165 microbial genomes (including bacterial, fungal and archaeal genomes) were downloaded from the NCBI RefSeq database. Then, built-in Perl scripts were used to sample restriction fragments from microbial genomes by each of 16 type 2B restriction enzymes, which formed a large 2bRAD microbial genome database. The set of 2bRAD tags sampled from each genome was assigned under the GCF number, as well as the GCF taxonomic information corresponding to the whole genome. Finally, all 2 bRAD tags from each GCF that occurred once within the genome were compared with those obtained from all the others. These 2bRAD tags are specific to a species-level taxon (without overlap with other species) and were developed as species-specific 2bRAD markers, collectively forming a 2bRAD marker database.

**Calculation of relative abundance using data obtained from 2bRAD-M sequencing**

First, to identify microbial species within each sample, all 2bRAD tags sequenced after quality control were mapped (using a built-in Perl script) against the 2bRAD marker database, which contains all 2bRAD tags theoretically unique to each of 26,163 microbial species in the database. To limit the number of false positives in the species identification, the G score was derived for each species identified within a sample using the method below, which is a harmonious mean of performing read coverage for 2bRAD markers belonging to a species and the number of all possible 2bRAD markers of this species. The threshold of the G score for false positive discovery of microbial species was set to 5 (41).

$${G score}_{species i}=\sqrt{S_{i}\times t_{i}}$$

Here, S represents the number of reads assigned to all 2bRAD markers belonging to species i within a sample and t represents the number of all 2bRAD markers of species i that have been sequenced within a sample. Then, the average read coverage of all 2 bRAD markers for each species was calculated, which represents the number of individuals belonging to a species detected in a sample at a given sequencing depth. The relative abundance of a given species was then calculated as the ratio of the number of microbial individuals belonging to a species to the total number of individuals from known species that can be detected within a sample.

$${Relative abundance}_{species i}=\frac{S_{i}/T_{i}}{\sum_{i=1}^{n} S_{i}/T_{i}}$$

Here, S represents the number of reads assigned to all 2bRAD markers of species i within a sample and T represents the number of all theoretical 2bRAD markers of species i.

Then, the 16S rRNA sequences corresponding to the species identified by 2bRAD-M were extracted, and the "Species <==> Functional" mapping. The “species <==> Function” mapping was weighted to obtain a functional abundance matrix. Differences in functional units in the functional abundance matrix were analyzed using ANOVA/Kruskal Wallis/T test/Wilcoxon algorithm
