# Supplementary material for: Gastrointestinal symptoms of long COVID-19 related to the ectopic colonization of specific bacteria that move between the upper and lower alimentary tract and alterations in serum metabolites
Source: BMC Med. 2023 Jul 19;21:264. doi: 10.1186/s12916-023-02972-x (PMC10355065; doi:10.1186/s12916-023-02972-x)
Supplement: Supplementary file 2 — Additional file 2: Table S1. Clinical characteristics of the enrolled patients for serum UPLC-MS/MS-based widely targeted metabolomics. [file 12916_2023_2972_MOESM2_ESM.docx]

**Table S1. Clinical characteristics of the enrolled patients for serum UPLC-MS/MS-based widely targeted metabolomics.**

| Characteristic | COVID-19 | Healthy Control |
| --- | --- | --- |
| ale, n (%) | 20(61) | 13(52) |
| Female, n (%) | 13(39) | 12(48) |
| Age, year | 44.2(23-68) | 35.6(23-56) |
| Hypertension | 6 | 3 |
| Diabetes mellitus | 4 | 2 |
| Hyperlipidaemia | 5 | 4 |
| Duration of hospitalization | 9.7(5-17) |  |
| Symptoms at admission, n (%)  Fever | 22(66.7) |  |
| Gastrointestinal symptoms, n (%)  Diarrhea | 5(15.5) |  |
| Respiratory symptoms, n (%) |  |  |
| Cough | 11(33.3) |  |
| Rhinorrhea (runny nose) | 18(54.5) |  |
| Gastrointestinal symptom of Long-COVID, n (%) |  |  |
| decreased appetite | 22(66.7%) |  |
| diarrhea | 12(36.3%) |  |
| xerostomia | 5(15.2%) |  |
| taste disorder | 2(6.1%) |  |
| emaciation | 4(12.1%) |  |
| abdominal pain | 1(3%) |  |
