# Supplementary material for: Gastrointestinal symptoms of long COVID-19 related to the ectopic colonization of specific bacteria that move between the upper and lower alimentary tract and alterations in serum metabolites
Source: BMC Med. 2023 Jul 19;21:264. doi: 10.1186/s12916-023-02972-x (PMC10355065; doi:10.1186/s12916-023-02972-x)
Supplement: Supplementary file 3 — Additional file 3: Figure S1. Diversity differences in the gut microbiome. Figure S2. Comparison of gut microbiota between recovers without GI symptoms at 3 months of follow-up after COVID-19 and those with mild disease. Figure S3. Diversity differences in the oral microbiome. Figure S4. Comparison of oral microbiota between recovers without GI symptoms at 3 months of follow-up after COVID-19 and those with mild disease. Figure S5. Alteration of specific metabolites in patient serum. [file 12916_2023_2972_MOESM3_ESM.docx]

**Additional file 3 Figure S1- Figure S5**


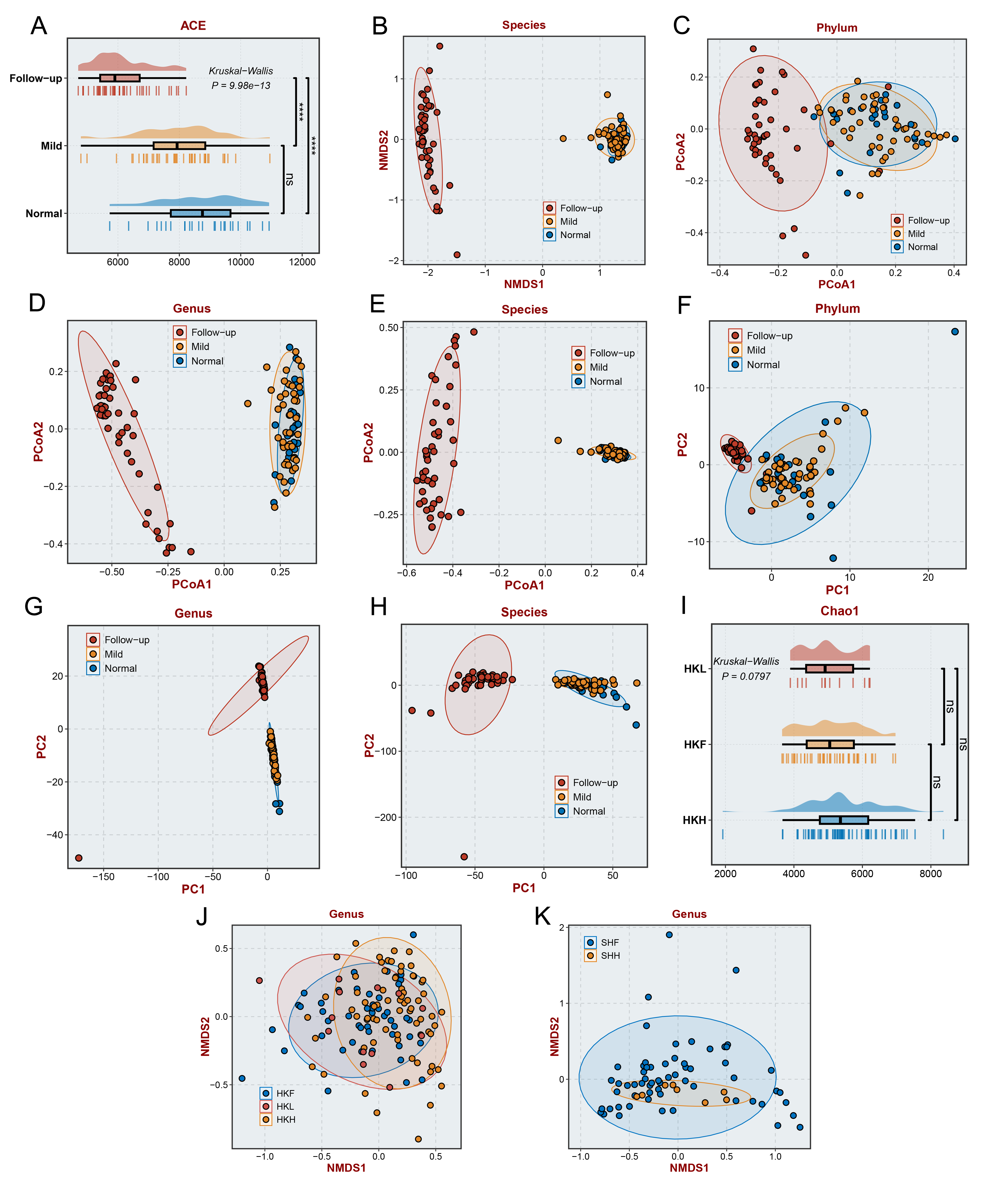


**Figure S1: Diversity differences in the gut microbiome. A.** The ACE index of the gut microbiome showed that the α diversity significantly decreased in the Follow-up group. ‘ns’ represents no significance, *p < 0.05, ** p < 0.01, ##p < 0.01, ***p < 0.001, and ****p < 0.0001(Student’t test).**B**. NMSD based on the relevant abundance of microbiome showed the composition of gut microbiota in the Follow-up group was conspicuously different from the Normal and Mild groups, at the species level. **C-H**. PCoA (**C-E**) and PCA (**F-H**) based on the relevant abundance of microbiome showed the composition of gut microbiota in the Follow-up group was conspicuously different from the Normal and Mild groups, both at the phylum and genus level. **I.** In the Hong Kong cohort, the Chao1 index of the gut microbiome showed that alpha diversity in the gut microbiome of COVID-19 patients followed up after discharge was not significantly different from that of the normal group and that of mild in-hospital patients. ‘ns’ represents no significance, *p < 0.05, ** p < 0.01, ##p < 0.01, ***p < 0.001, and ****p < 0.0001(Student’t test). **J.** In the Hong Kong cohort, genus-level NMDS analysis could not distinguish among the three groups based on the relative abundance of gut microbiota. **K.** In the Shanghai cohort, genus-level NMDS analysis suggested that the relative abundance of gut microbiota could not distinguish between the mild COVID-19 patients and the normal.


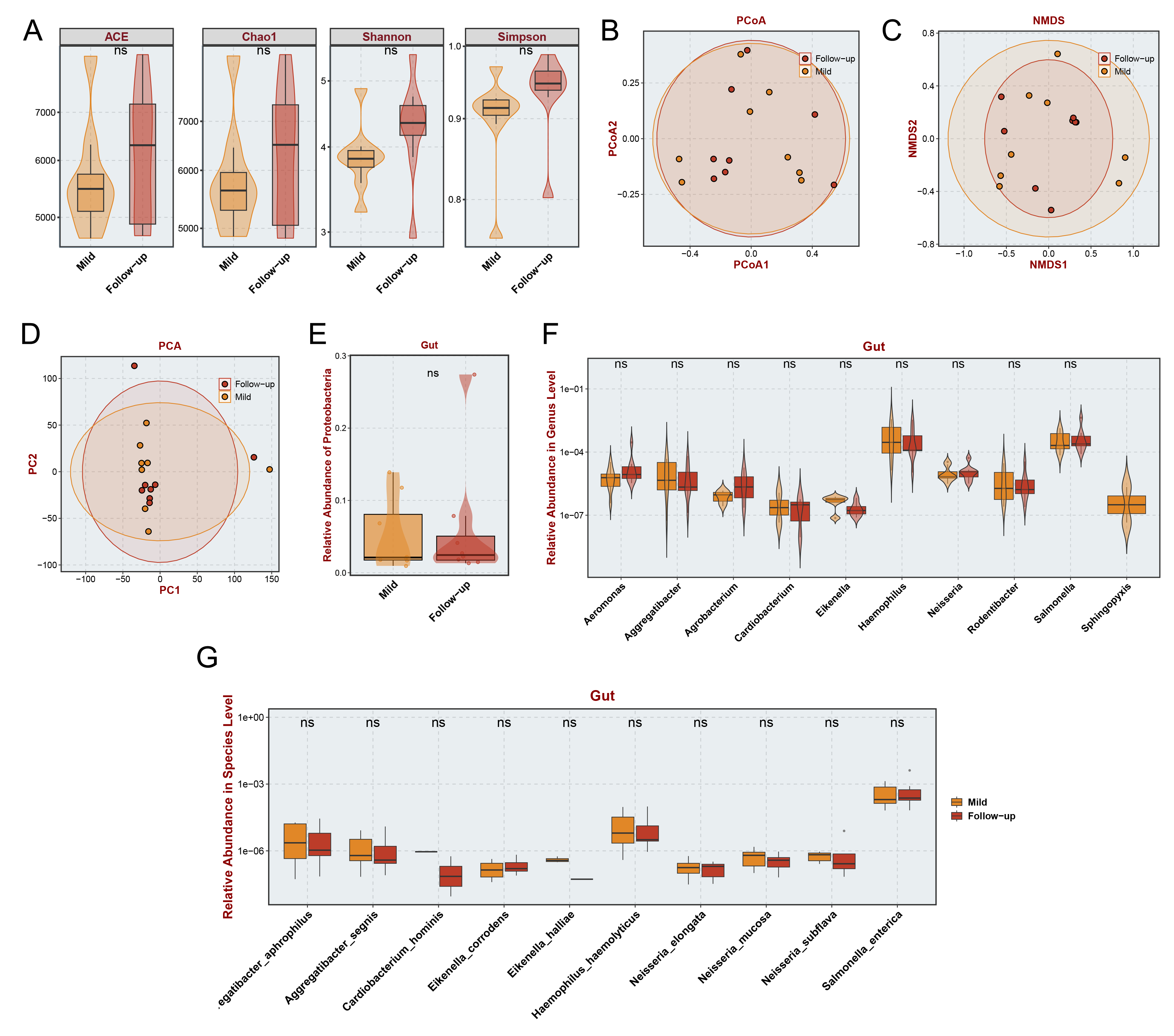


**Figure S2: Comparison of gut microbiota between recovers without GI symptoms at 3 months of follow-up after COVID-19 and those with mild disease. A.** Comparison of Alpha diversity about gut metagenome between mild patients and recovers without GI symptoms, including ACE, Chao1, Shannon, and Simpson index. **B-D.** Comparison of Beta diversity about gut metagenome with PCoA (B), NMDS (C), and PCA (D) anlaysis, between Mild and Follow-up group. **E-G.** To compare the difference of GI symptoms-associated differential flora in recovers without GI symptoms from phylum (E), genus (F), and species (G) level in gut. Follow-up (n = 8): recovers without GI symptoms at 3 months of follow-up after COVID-19; Mild (n = 8): Patients with confirmed mild COVID-19. ‘ns’ represents no significance.


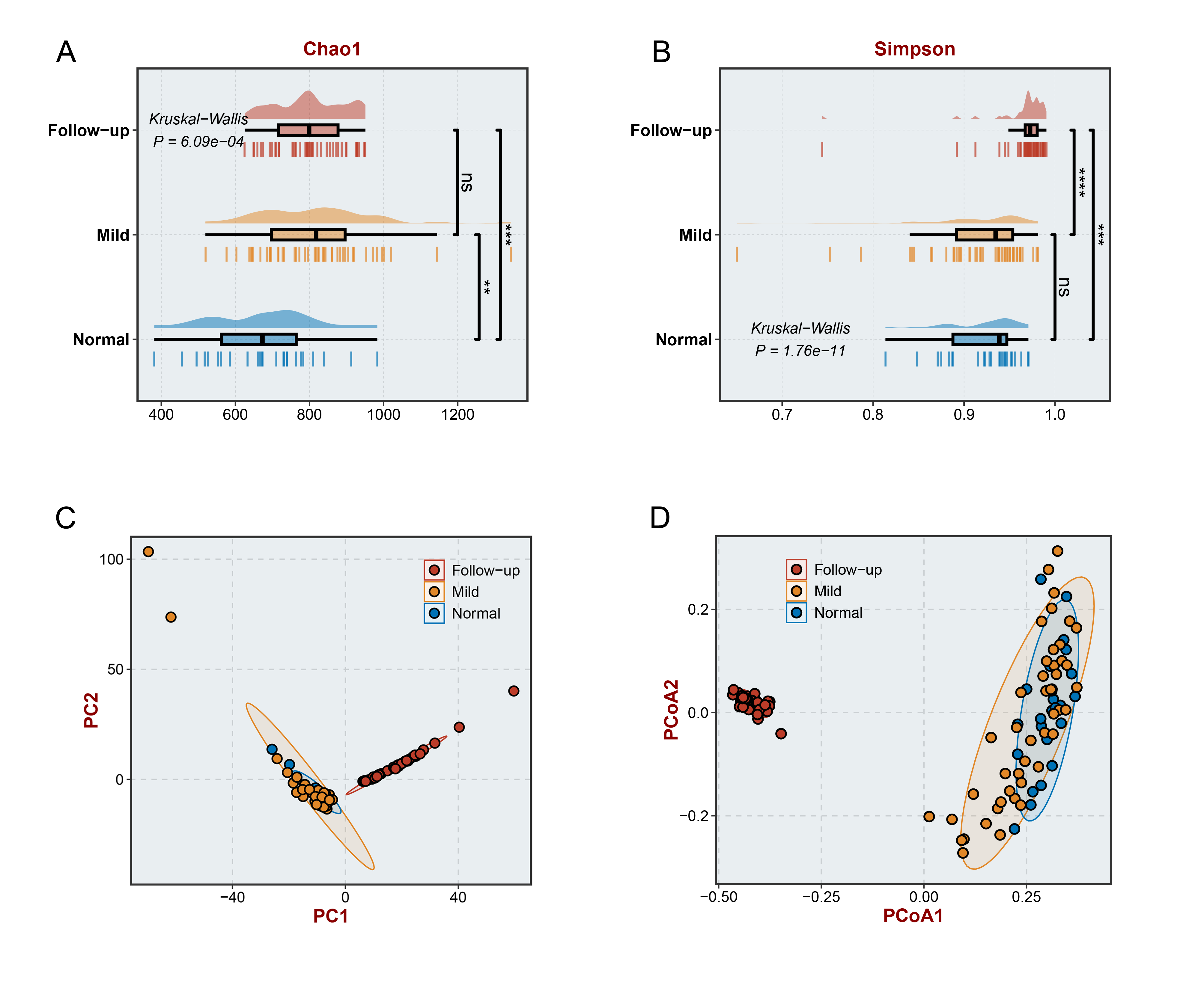


**Figure S3: Diversity differences in the oral microbiome**. **A-B.** The Chao1 (**A**) and Simpson (**B**) index of the oral microbiome showed that the alpha diversity significantly increased in the Follow-up group. ‘ns’ represents no significance, *p < 0.05, ** p < 0.01, ##p < 0.01, ***p < 0.001, and ****p < 0.0001(Student’t test). **C-D.** Based on the relevant abundance of the microbiome, the PCoA (**C**) and PCA (**D**) showed that the composition of oral microbiota in the Follow-up group was conspicuously different from the Normal and Mild groups.


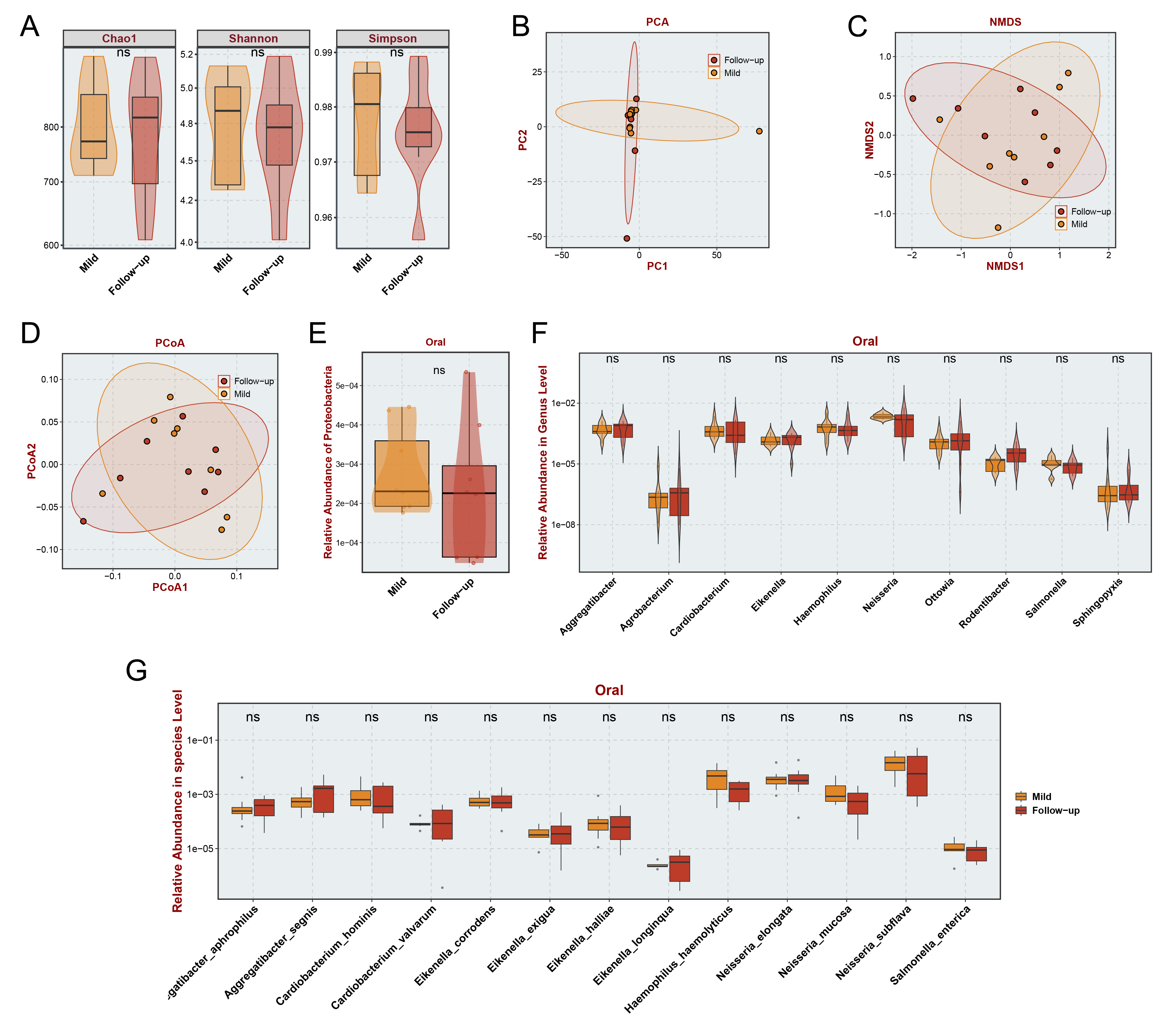


**Figure S4: Comparison of oral microbiota between recovers without GI symptoms at 3 months of follow-up after COVID-19 and those with mild disease. A.** Comparison of Alpha diversity about oral flora between mild patients and recovers without GI symptoms, including Chao1, Shannon, and Simpson index. **B-D.** Comparison of Beta diversity about oral flora with PCA (B), NMDS (C), and PCoA (D) anlaysis, between Mild and Follow-up group. **E-G.** To compare the difference of GI symptoms-associated differential flora in recovers without GI symptoms from phylum (E), genus (F), and species (G) level in oral. Follow-up (n = 8): recovers without GI symptoms at 3 months of follow-up after COVID-19; Mild (n = 8): Patients with confirmed mild COVID-19. ‘ns’ represents no significance.


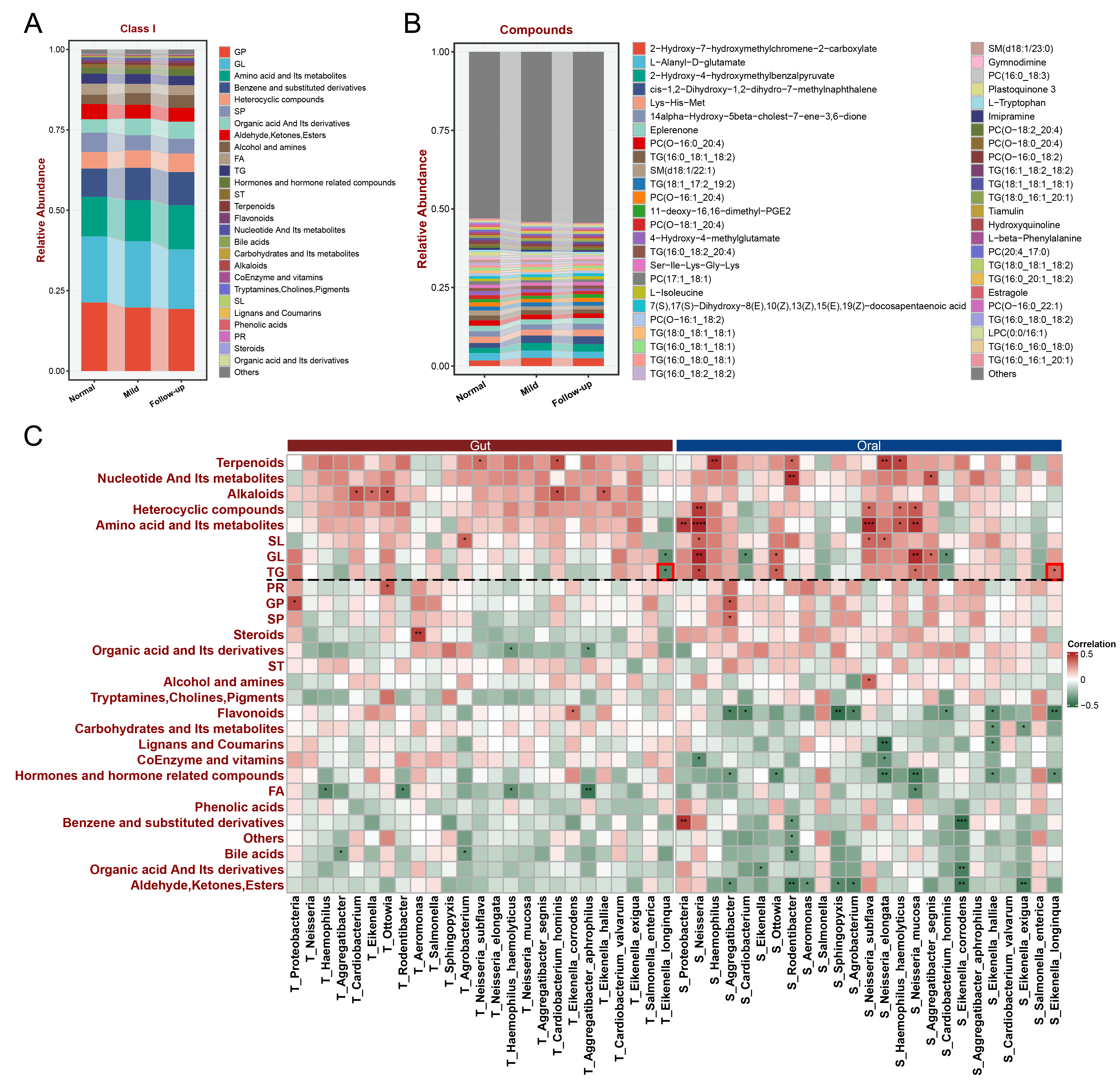


**Figure S5: Alteration of specific metabolites in patient serum.** **A-B.** Average relative abundance of top 27 Class I metabolites (A) and top 49 compounds (B) in serum from the normals, in-hospital patient, and their follow-up within three months after discharge. (C) Association analysis between the abundance of significantly altered metabolites in serum and altered abundance of digestive tract microbiomes in specie level in the follow-up group. ‘ns’ represents no significance, *p < 0.05, ** p < 0.01, ***p < 0.001, and ****p < 0.0001(Student’t test).
