# Supplementary material for: Gastrointestinal symptoms of long COVID-19 related to the ectopic colonization of specific bacteria that move between the upper and lower alimentary tract and alterations in serum metabolites
Source: BMC Med. 2023 Jul 19;21:264. doi: 10.1186/s12916-023-02972-x (PMC10355065; doi:10.1186/s12916-023-02972-x)
Supplement: Supplementary file 4 — Additional file 4: Table S2. Differential metabolites in serum between follow-up group and mild group. [file 12916_2023_2972_MOESM4_ESM.docx]

**Table S2. Differential metabolites in serum between follow-up group and mild group. (VIP ≥1, logFC ≥1.5 or ≤-1.5, p value ≤0.05)**

| **Follow-up group VS. Mild group** | | | |
| --- | --- | --- | --- |
| **Compounds** | **Log2FC** | **P-value** | **VIP** |
| Bisphenol A diglycidyl ether | -9.150215767 | 3.03E-09 | 3.03607218 |
| PA(21:0_24:1) | -8.084750361 | 1.78E-16 | 4.253114834 |
| Hexadecylamine | -7.171400141 | 0.00179329 | 1.261240581 |
| 2'-Deamino-2'-hydroxyneamine | -5.682270143 | 4.01E-09 | 3.090037744 |
| Combrestatin A4 | -5.318676731 | 0.001917219 | 1.20237989 |
| Octadecylamine | -4.881145517 | 0.002034941 | 1.546232703 |
| Arg-Gln-Arg | -4.194970063 | 4.83E-09 | 3.75107108 |
| Prephenic acid | -2.831645351 | 0.000169748 | 2.162925139 |
| ESTRA-4,9-DIENE-3,17-DIONE | -2.455436064 | 5.74E-23 | 4.034773108 |
| 4-toluenesulfonic acid | -2.171854424 | 4.24E-08 | 3.342615142 |
| DL-Stachydrine | -2.15036528 | 1.07E-06 | 2.787282983 |
| N,N-Bis(2-hydroxyethyl)dodecanamide | -2.122434125 | 1.71E-10 | 3.305617558 |
| 2'-Deoxycytidine-5'-triphosphate | -1.681114077 | 1.26E-09 | 2.815128292 |
| 15(R)-Prostaglandin E1 | -1.614813887 | 0.006007303 | 1.65142735 |
| Undecanoylcholine | -1.589197723 | 4.35E-09 | 2.855192927 |
| Resorcinolnaphthalein | -1.561858799 | 1.66E-07 | 2.835511863 |
| Tyr-Phe4Cl-OH | -1.533301091 | 0.049305639 | 1.610107662 |
| Psoromic Acid | -1.517504693 | 4.21E-06 | 1.962262898 |
| 10-Deoxymethynolide | 1.51006056 | 0.008110574 | 2.077067404 |
| 3-Nitrobenzanthrone | 1.518721673 | 0.003929593 | 1.143909196 |
| Ser-Tyr-Tyr-Gln-Ser | 1.528806421 | 5.59E-05 | 2.456013234 |
| Amlodipine | 1.688348793 | 0.028625175 | 1.823458671 |
| 4-Chlorophenylacetic acid | 1.809466773 | 0.016420806 | 1.482654938 |
| 5-Sulfoxymethylfurfural | 1.818381636 | 0.015074159 | 1.525122958 |
| LPC(12:0/0:0) | 1.859292536 | 0.006562539 | 1.363320132 |
