# Supplementary material for: Gastrointestinal symptoms of long COVID-19 related to the ectopic colonization of specific bacteria that move between the upper and lower alimentary tract and alterations in serum metabolites
Source: BMC Med. 2023 Jul 19;21:264. doi: 10.1186/s12916-023-02972-x (PMC10355065; doi:10.1186/s12916-023-02972-x)
Supplement: Supplementary file 5 — Additional file 5: Table S3. Differential metabolites in serum between follow-up group and normal group. [file 12916_2023_2972_MOESM5_ESM.docx]

**Table S3. Differential metabolites in serum between follow-up group and normal group. (VIP ≥1, logFC ≥1.5 or ≤-1.5, p value ≤0.05)**

| **Follow-up group VS. Normal group** | | | |
| --- | --- | --- | --- |
| **Compounds** | **P-value** | **Log2FC** | **VIP** |
| DL-Benzylsuccinic acid | 1.98E-05 | -1.54272702 | 1.913766291 |
| 4,4'-Dihydroxydiphenylmethane | 6.68E-06 | -1.821122637 | 1.99619987 |
| 2,2'-Methylenediphenol | 3.38E-06 | -1.839606864 | 1.969099564 |
| Plastoquinone 3 | 1.78E-07 | -1.654669789 | 1.930821775 |
| Acerogenin G | 1.21E-06 | -1.9444412 | 1.99515798 |
| Estradiol valerate | 5.30E-06 | -1.557727787 | 1.927629943 |
| 2,6-Dimethylhexestrol | 1.36E-06 | -1.896632465 | 2.015933375 |
| Cannabidiolic acid | 3.36E-07 | -1.715370569 | 1.940267852 |
| Jervine | 1.02E-09 | -2.309168257 | 2.144624076 |
| 13-Hydroxy-9-methoxy-10-oxo-11-octadecenoic acid | 3.09E-06 | -1.643456568 | 1.938431224 |
| PA(21:0_24:1) | 2.85E-11 | -8.643644489 | 2.825879995 |
| 2,4-diacetamino-2,4,6-triphenoxy-D-mannopyranose | 6.07E-07 | 1.91519306 | 1.921908718 |
| (±)12-HETE | 8.36E-06 | 2.657410287 | 1.707902269 |
| N-Caffeoyl-Putrescine | 6.70E-08 | 6.993512342 | 1.956238453 |
| L-Tryptophanamide | 9.79E-23 | 1.798238205 | 2.733060989 |
| Delta-Hexalactone | 3.18E-21 | 2.137009751 | 2.5886324 |
| Carnitine ph-C14 | 1.19E-05 | 1.798539784 | 1.461643364 |
| Carnitine C6:DC | 0.03935004 | 1.527670488 | 1.054002756 |
| Leu-Leu-Glu | 2.90E-07 | 5.06097069 | 1.741643795 |
| N-(1-Deoxy-1-fructosyl)phenylalanine | 1.80E-06 | 2.436391109 | 1.709454154 |
| Ile-Asp | 1.08E-06 | 1.71864662 | 1.916486538 |
| Hexaethylene-glycol | 1.83E-07 | 3.385377851 | 1.6438654 |
| 3,6,9,12,15,18,21-Heptaoxatricosane-21,23-diol | 6.78E-08 | 5.621137289 | 1.751906118 |
| Hexadecylamine | 0.00858157 | 3.259333507 | 1.104587305 |
| Tetraethylene-glycol | 6.66E-08 | 10.81395853 | 1.838035277 |
| Asp-Leu | 1.08E-06 | 1.71864662 | 1.916486538 |
| Asp-Ile | 1.08E-06 | 1.71864662 | 1.916486538 |
| Heptethylene-glycol | 7.59E-08 | 7.332947517 | 1.662096103 |
| 4-Hydroxyquinoline | 3.10E-23 | 1.509642099 | 2.651776102 |
| Troglitazone | 8.71E-08 | 10.38047227 | 1.754933557 |
| 1,5-Dihydroriboflavin 5'-(dihydrogen phosphate) | 1.08E-07 | 4.995149878 | 1.733273339 |
| 21-hydroxy-heneicosanoic acid | 4.24E-10 | 1.532570372 | 2.025159359 |
| Solavetivone | 4.66E-08 | 2.104491752 | 1.874343988 |
| Gly-Leu-Arg-Val-Phe | 1.39E-07 | 4.062274122 | 1.469686289 |
| D-Xylono-1,4-lactone | 2.59E-08 | 1.833548411 | 2.328411275 |
| Dofetilide | 7.04E-08 | 10.36713128 | 2.018105886 |
| Glucoalyssin | 2.00E-07 | 5.712027113 | 1.687568543 |
| Cys-Gln-Met | 1.73E-07 | 7.272930363 | 2.018995299 |
| Val-Ile-Leu-Asp | 9.62E-08 | 4.733460091 | 1.511794221 |
| 4-Chlorophenylacetic acid | 0.008336755 | 2.249162887 | 1.291946484 |
| Arg-Thr-Ala-Arg | 1.21E-07 | 4.286790066 | 1.479557199 |
| Westiellamide | 1.29E-07 | 4.191977001 | 1.489521181 |
| Val-Ala-Phe-Asp | 8.35E-08 | 6.075696272 | 1.573855058 |
| Ser-Tyr-Tyr-Gln-Ser | 5.96E-06 | 1.918855715 | 1.861272682 |
| Gln-Asn-Leu-Glu | 1.00E-07 | 4.556812933 | 1.507713219 |
| 7-Oxocallitrisic acid,methyl ester | 7.24E-08 | 6.346231098 | 1.737695144 |
| Atrovirisidone | 5.45E-08 | 6.021568331 | 2.039723538 |
| Rivaroxaban | 8.10E-08 | 2.39912755 | 1.656940778 |
| HC Toxin | 1.04E-07 | 5.287099206 | 1.584776126 |
| Penoxsulam | 9.07E-08 | 5.046458702 | 1.806852894 |
| Tyr-Cys-Trp | 3.45E-06 | 1.514310962 | 1.734049681 |
| N-Acetylsulfadiazine | 7.89E-08 | 7.223516823 | 1.667085629 |
| Ajmaline | 7.11E-08 | 6.979654791 | 1.670715873 |
| Ala-Ala-Ala-Arg-Phe | 4.53E-07 | 1.835839686 | 1.757697401 |
| Ala-Thr-Ile-Lys | 8.24E-08 | 5.397087875 | 1.552495689 |
| Thr-Glu-Leu-Lys | 1.41E-06 | 3.362380414 | 1.591371932 |
| Pyridoxamine 5-phosphate | 6.25E-08 | 7.751340836 | 1.709135874 |
| Phe4Cl-Met-OH | 0.044238137 | 1.994492718 | 1.190049569 |
| Yuzu lactone | 0.019388819 | 1.745763958 | 1.672407715 |
| Ala-Asp-Arg-Arg | 2.77E-07 | 3.321355588 | 1.708081652 |
| Ala-Gly-Leu-Val-Ser | 3.98E-07 | 3.522840485 | 1.577701059 |
| Thr-Val-Ala-Lys | 7.32E-08 | 5.398960745 | 1.889314932 |
| 2-Phenylpropanal | 2.61E-07 | 3.291140884 | 1.705681861 |
| Zoledronic acid | 3.53E-20 | 1.549884984 | 2.578469382 |
| Tralkoxydim | 2.65E-08 | 4.772882212 | 2.176820424 |
| 5-Sulfoxymethylfurfural | 0.007131575 | 2.293062743 | 1.335337262 |
| Antanapeptin C | 1.09E-06 | 2.686055314 | 1.398278543 |
| Azobenzene | 1.02E-07 | 8.239803885 | 1.767271973 |
| Tolbutamide | 7.91E-08 | 5.723901775 | 1.446024029 |
| 1-(4-Methylphenyl)acetone | 7.04E-07 | 2.463319886 | 1.223821999 |
| Glu-Arg-Glu | 2.03E-07 | 7.805804664 | 1.970093433 |
| Trilostane | 6.90E-08 | 3.093446128 | 1.683546529 |
| Alpha-Pyrrolidinopropiophenone | 8.91E-08 | 6.30019831 | 1.637903918 |
| Daunorubicin | 2.03E-07 | 9.140838639 | 1.844617234 |
| (S)-2-Hydroxy-2-methylsuccinic acid | 1.47E-15 | 2.516948896 | 2.684292977 |
| Gln-Trp-Trp | 1.73E-07 | 8.057980225 | 1.789027495 |
| 1,2-Dimethoxybenzene | 9.71E-08 | 5.072476947 | 1.612208159 |
| Dehydrocostus Lactone | 6.69E-08 | 10.66831643 | 1.835688353 |
| Rutin | 5.38E-08 | 7.432816117 | 2.151029581 |
| Mebhydrolin | 6.89E-08 | 8.561104975 | 1.620087922 |
| 2,6-Dihydroxyanthraquinone | 1.82E-07 | 4.075538193 | 1.419420723 |
| Lys-Phe-Asp-Lys | 1.87E-07 | 4.535719928 | 1.575278496 |
| Salmeterol | 8.51E-08 | 5.647303569 | 1.449495796 |
| 4-Hydroxyindole | 1.18E-07 | 8.479619742 | 1.868997536 |
| Raloxifene | 8.54E-08 | 8.148332635 | 1.943858156 |
| Thr-Tyr-His | 9.71E-08 | 6.147284833 | 1.433840136 |
| Heptenophos | 5.01E-30 | 2.14741672 | 2.691536475 |
| Isosyringinoside | 1.82E-07 | 4.21912652 | 1.786343139 |
| Varanic acid | 1.30E-07 | 5.468673395 | 1.733510019 |
| LPG(22:5/0:0) | 1.79E-15 | 8.946015357 | 2.816231469 |
| LPS(18:0/0:0) | 3.48E-08 | 1.628349861 | 1.971772096 |
| LPS(20:4/0:0) | 8.14E-10 | 9.550649355 | 2.776592636 |
| LPA(0:0/22:6) | 1.72E-06 | 1.598186512 | 1.632275751 |
| LPG(18:3) | 2.16E-12 | 8.990592134 | 2.817094218 |
| LPG(22:4) | 2.14E-12 | 9.164333204 | 2.807790809 |
| CE(23:0) | 3.15E-18 | 8.745334984 | 2.833371212 |
